# Multi-omics revealed the effects of rumen to blood path on early lactation performance in transition dairy cows

**DOI:** 10.1186/s40168-026-02403-y

**Published:** 2026-04-07

**Authors:** Guangfu Tang, Chenguang Zhang, Xia Zhang, Huifeng Liu, Garret Suen, Junhu Yao, Jun Zhang

**Affiliations:** 1 College of Animal Science and Technology, Northwest A&F University, Yangling, 712100 China; 2 College of Veterinary Medicine, Northwest A&F University, Yangling, 712100 China; 3 Department of Bacteriology, University of Wisconsin-Madison, Madison, WI 53706 USA

**Keywords:** Transition period, Milk production, Dairy cow, Ruminal metagenome, Metabolome, Structural equation model, Pan-genome

## Abstract

**Background:**

The transition period is vitally important to the life cycle of dairy cows. However, the function of the microbiota during both pre- and post-partum and their relationship with ruminal, plasma, and milk metabolites still require systematic investigation. To address this, the 7 highest- and 7 lowest-performing animals among a cohort of 100 dairy cows were selected based on their postpartum energy-corrected milk yield. Rumen fluid and plasma samples were collected during both pre- and post-partum periods, whereas milk samples were obtained postpartum. Shotgun metagenomics of rumen contents in addition to metabolomics of rumen, plasma, and milk samples were performed to evaluate the associations between ruminal microbes and early lactation performance in transition dairy cows.

**Results:**

Compared with prepartum cows, postpartum high-yield cows had greater concentrations of ruminal volatile fatty acids and plasma total bile acid. Moreover, plasma urea nitrogen and most amino acids, peptides, and their derivatives in plasma and milk were increased in postpartum high-yield cows, relative to postpartum low-yield cows. Metagenomic analysis revealed that the relative abundances of several species within the *Prevotella*, *Succinimonas*, *Succinatimonas*, and *Methanosphaera* increased, while other bacteria belong to *Alistipes* and *Bacteroides*, and archaeal *Methanobrevibacter* species decreased in postpartum cows, particularly in postpartum high-yield cows. Co-occurrence network and correlation analysis suggested that *Prevotella* and *Succinatimonas* were negatively correlated to *Alistipes*, *Bacteroides*, and *Methanobrevibacter*, potentially contributing to the nutritionally efficient phenotype of postpartum high-yield cows. A metabolic pathway analysis of our metagenomic data revealed that postpartum high-yield cows possessed more microbial genes involved in starch utilization and amino acid synthesis, while a wide range of microbial genes involved in cellulose utilization, acetogenesis, and amino acid degradation were found in prepartum cows with low-yield in postpartum. A structural equation model analysis showed that the increased relative abundances of *Prevotella tf.2–5* and *Succinatimonas CAG_777* were related to greater concentrations of plasma chenodeoxycholic acid glycine conjugate, milk 5-Methoxytryptophan, and energy-corrected milk yield. Finally, pan-genomic analysis confirmed that *Alistipes*, *Bacteroides*, and *Methanobrevibacter* possess genetic conservation of both hydrogenases and dehydrogenases, which may contribute to energy loss in the rumen via hydrogen dissipation.

**Conclusion:**

In summary, our findings provide a fundamental understanding of how microbiome-dependent mechanisms contribute to early lactation performance in dairy cows during the transition period. The increased abundance of *Prevotella*, *Succinimonas*, and *Succinatimonas* in postpartum cows suggest that they are important microbes during the transition period and may help in coping with metabolic challenges, while improving nutrient utilization efficiency during this period. Our study underscores the importance of the ruminal microbiome during the transition period and highlights the need for rumen-based nutritional intervention strategies to improve production efficiency in ruminants.

Video Abstract

**Supplementary Information:**

The online version contains supplementary material available at 10.1186/s40168-026-02403-y.

## Introduction

The transition period (3 weeks before and after calving) is vitally important in the life-cycle of dairy cows. During this period, cows experience numerous environmental and physiological changes, including calving and the initiating of lactation [1], switching to a high-energy diet [2], alterations in hormone levels [3], and varied feeding behaviors [4]. These changes can greatly impact nutrient acquisition and energy partitioning, thereby imposing significant metabolic stress and challenges during early lactation. When cows are unable to cope with these stressors, they can develop a variety of metabolic diseases including ketosis, fatty liver, and displacement of the abomasum. The occurrence of these metabolic diseases during the transition period has been correlated with a reduction in milk production and an extended interval between calving and conception [5–7]. Therefore, maintaining the health of dairy cows throughout the transition period is important for ensuring their sustainable economic benefits.

As the first compartment of the gastrointestinal tract, the rumen, and its homeostatic maintenance, are crucial for nutrient supply and the overall health of the animal. Importantly, volatile fatty acids (VFAs) derived from ruminal microbes can supply more than 70% of the energy requirements for adult ruminants [8]. Recent studies have focused on delineating the specific roles of the rumen and its symbiotic microbiota. During the transition period, changes in the rumen microbial structure of cows are dramatic. Notably, numerous studies have confirmed that the rapid postpartum increase in *Prevotella* represents a distinctive characteristic of the ruminal microbiota in dairy cows under high-fiber/high-starch feeding regimens [9–11]. In addition, changes in rumen archaeal dynamics are also noteworthy, with some genera, such as *Methanobrevibacter*, exhibiting decreased relative abundances, while others, such as *Methanosphaera* and *Methanomassiliicoccus* are known to increase in relative abundance postpartum [11–13]. However, the comprehensive impact of these microbial dynamics on both the ruminal environment and physiology of the dairy cow remains poorly understood.

Although mechanistic insights remain limited, certain rumen microbial taxa have been empirically associated with metabolic outcomes in transition dairy cows. For instance, recent investigations revealed that members of the Christensenellaceae, Ruminococcaceae, Lachnospiraceae, and Prevotellaceae were significantly enriched in the ruminal microbiome of dairy cows with ketosis [14]. A dataset comprising 61 ketosis cows and 84 healthy controls revealed that *Prevotella* in the rumen may elevate blood glucose levels and reduce β-hydroxybutyrate concentrations by modulating the production of gluconeogenic amino acids and propionate [15]. Similarly, Kong et al. [16] discovered that *Ruminococcus bovis* is associated with host energy metabolism homeostasis by supplying glucogenic precursors to the liver. The lactation curve of dairy cows demonstrates that milk production begins rapidly after calving and quickly peaks during the lactation cycle [17]. Previous studies have predominantly focused on the ruminal microbiota during peak or mid-to-late lactation stages [18–20]. Although previous studies have examined the links between ruminal microbiota and lactation traits in the transition dairy cows [21, 22], our understanding of the functions of these microbes during the pre- and postpartum periods, as well as their relationships with ruminal, plasma, and milk metabolites, remains limited. To address this, we employed ruminal shotgun metagenomics and metabolomics of ruminal, plasma, and milk samples obtained from a cohort of 14 dairy cows exhibiting high and low milk yield during the transition period. The potential influence of ruminal microbes on milk production performance during this period was investigated. This study will provide fundamental information about the microbiome-dependent mechanisms that contribute to high milk production in postpartum dairy cows.

## Materials and methods

### Animals, sampling, and grouping

One hundred healthy multiparous Chinese Holstein dairy cows with similar milk yields (from the last 305 days), parity, body condition scores, and calving due dates were selected from a large cohort of 3,000 dairy cows at the Modern Farming (Saibei) Co., Ltd. (Zhangjiakou, China). All dairy cows were housed in a free-stall barn and had ad libitum access to water with a total mixed ratio (TMR) provided daily at 06:30, 12:30, and 19:30 h. The ingredients and chemical composition of the TMR provided at prepartum and postpartum is presented in Supplemental Table S1. After calving, cows were milked three times daily at 07:00, 13:00, and 21:00 h.

Rumen fluid from all cows was collected by a flexible esophageal tube (2 mm of wall thickness and 6 mm of internal diameter; Anscitech Co., Ltd., Wuhan, China) at 14 d prior to and 14 d after calving at 2 h after morning feeding. For each sampling, the first 50 mL of liquid was discarded to prevent salivary contamination. The liquid fraction was obtained by filtering rumen fluid through 4 layers of cheesecloth. Blood samples were collected from the caudal vein of each cow prior to rumen fluid sampling with EDTA as an anticoagulant. After setting at room temperature for about 30 min, plasma was obtained by centrifuged at 3,000 × *g* for 10 min. Milk samples consisting of 1/3 from the morning, 1/3 from the afternoon, and 1/3 from the evening milking were pooled as a daily sample of 60 mL from all cows at 13–14 d. All samples used for omics analysis were flash frozen in liquid nitrogen, and then transferred to − 80℃ until downstream analyses. Daily milk samples for composition analysis were preserved with potassium dichromate and stored at 4℃ for further measurements.

Average milk yield determined at 13 d, 14 d, and 15 d after calving were used to calculate energy-corrected milk (ECM) for all cows according to NRC guidelines [23] as follows: ECM kg/d = [(0.3246 × milk yield) + (12.86 × fat yield) + (7.04 × protein yield)]. According to the ECM yield of all cows over these three days, the 7 cows (parity: 2.57 ± 0.53, body weight: 671.57 ± 69.75 kg, mean ± standard deviation) with the highest ECM (51.07 ± 7.76 kg/d) and the 7 cows (parity: 2.14 ± 0.38, body weight: 642.71 ± 76.10 kg) with the lowest ECM (31.19 ± 3.36 kg/d) were selected and used for our study (Fig. S1). When considering both sampling time points (pre- and post-partum) and ECM, the rumen and blood samples from these 14 cows were divided into four groups: (1) prepartum cows with high ECM yield in postpartum (PREP_H, *n* = 7); (2) prepartum cows with low ECM yield in postpartum (PREP_L, *n* = 7); (3) postpartum cows with high ECM yield (POSP_H, *n* = 7); and (4) postpartum cows with low ECM yield (POSP_L, *n* = 7). When only considering the sampling time points, we divided samples into two groups: prepartum (PREP, *n* = 14) and postpartum (POSP, *n* = 14).

### Rumen fermentation parameters analysis

Rumen fluid pH was measured with a mobile pH meter (pH818; Wanchuang Electronic Products Co., Ltd., Dongguan, China) immediately after sampling. Thawed rumen fluid samples were centrifuged at 12,000 × *g* at 4℃ for 10 min to obtain a clear supernatant. A total of 1 mL supernatant was added with 100 µL of 25% metaphosphoric acid and used to determine VFAs according to a previously described method [24]. Another 1 mL supernatant was analyzed for NH_3_-N using a phenol-hypochlorite assay [25]. Ruminal lactate concentration was determined using a commercial kit according to the manufacturer's instruction (cat. #A019-2-1; Nanjing Jiancheng Bioengineering Institute, Nanjing, China).

### Plasma individual metabolites and milk composition analysis

Plasma total bile acids (total BA; cat. #TBA7080), total protein (cat. #TP7080), albumin (cat. #ALB7080), glucose (cat. #GLU7080), total cholesterol (cat. #TC7080), alanine aminotransferase (ALT; cat. #ALT7080), alkaline phosphatase (cat. #ALP7080), and urea nitrogen (BUN; cat. #UR7080) were determined using commercial kits (Hunan Yonghe-Sun Biotechnology Co., Ltd., Changsha, China) in a clinical autoanalyzer (Celercare V5; Tianjin MNCHIP Technologies Co., Ltd, Tianjin, China). The concentration of globulin was calculated by subtracting the concentration of albumin from the concentration of total protein. The concentration of plasma beta-hydroxybutyric acids was measured by a commercial kit according to the manufacturer's instruction (cat. #E030-1-1; Nanjing Jiancheng Bioengineering Institute, Nanjing, China). Fresh milk samples were analyzed for fat, protein, and lactose contents using a milk composition analyzer (MilkoScan FT1, FOSS, Denmark).

### Rumen metagenome sequencing and data processing

Total DNA was extracted from rumen fluid samples by using an E.Z.N.A. stool DNA kit (cat. #D4015; Omega, Inc., USA) with the repeat bead-beating step plus column method [26]. The quality of DNA was visually checked by 1% agarose gel electrophoresis and the DNA concentration was determined using a NanoDrop 2000 spectrophotometer (Thermo Fisher Scientific, Waltham, USA). Each DNA extract was fragmented to an average size of approximately 350 bp using a Covaris M220 (Gene Company Limited, China) for paired-end library construction. Sequencing libraries were prepared using the TruSeq™ DNA Sample Prep Kit (Illumina, San Diego, CA, USA). A 2 × 150 bp paired-end sequencing was performed on an Illumina NovaSeq (Illumina Inc., San Diego, CA, USA) with a NovaSeq 6000 S4 Reagent Kit v1.5 (Illumina Inc., San Diego, CA, USA) according to the manufacturer's instructions.

After sequencing, adapter sequences were trimmed from the paired-end reads using SeqPrep (v1.1, https://github.com/jstjohn/SeqPrep) and low-quality reads were removed using Sickle (v1.33, https://github.com/najoshi/sickle). Host reads were removed by aligning reads against the *Bos taurus* genome with BWA (v0.7.17) [27] and filtered. The remaining reads were de novo assembled into contigs using Megahit (v1.1.2) [28] and open reading frames (ORFs) were predicted using MetaGeneMark (v2.10) [29]. All ORFs sharing ≥ 95% sequences identity over ≥ 90% of their length were clustered using CD-HIT (v4.6.1) [30]. High-quality reads were mapped to the representative sequences with 95% identity and to calculate the abundances of microbial genes (reads per kilobase per million mapped reads, RPKM) using SOAPaligner (v2.2.1) [31]. Representative sequences of non-redundant gene catalog in our metagenomes were aligned to the NCBI's nr database using Diamond (v0.8.35) for taxonomic annotations using the Best-hit method [32]. Functional profiling of the metagenome was performed using Diamond alignment against the Kyoto Encyclopedia of Genes and Genomes database (KEGG v94.2), which enables systematic interpretation of biological systems to elucidate the functions of the microbiome in the host [32]. The abundances of KEGG enzymes were normalized to RPKM for subsequent analysis of metabolic pathways. Microbial beta diversity was determined using the distance matrices generated from a Bray–Curtis based, non-metric multidimensional scaling analysis (NMDS), and an analysis of similarities (ANOSIM) [33].

### Untargeted metabolomics analysis

Rumen fluid, plasma, and milk samples were subjected to metabolomic analysis using a liquid chromatograph-mass spectrometer system (LC–MS, Q-Exactive, Thermo Fisher Scientific, USA) combined with a quadrupole-time-of-flight mass spectrometer (Triple TOF™ 5600 +, AB Sciex, USA) equipped with an electrospray ionization source operating in both positive and negative modes. Briefly, the metabolites were extracted using methanol/acetonitrile (1:1, v/v) buffer at a 1:2 ratio of sample:buffer. The mixture was allowed to settle at −20℃ and treated using a high-throughput tissue crusher (Wonbio-96C, Shanghai Major biotechnology Co., LTD) at 50 Hz for 6 min followed by vortex for 30 s and ultrasound at 40 kHz for 30 min at 5℃. The samples were placed at −20℃ for 30 min to precipitate proteins, and then were centrifuged at 13,000 × *g* at 4℃ for 15 min to obtain a supernatant. As part of the system conditioning, a quality control process was performed on a pooled quality control sample by mixing equal volumes (20 μL) of each sample.

Chromatographic separations were performed on an ACQUITY UPLC HSS T3 column (100 mm × 2.1 mm, 1.8 μm; Waters, Milford, USA). The LC–MS data were processed using Progenesis QI software (Nonlinear Dynamics, Waters, USA) to extract raw peaks, filter and calibrate baseline, align peaks, deconvolute, identify peaks, and integrate peak areas. The unidentified peaks and metabolite peaks that presented in < 50% of samples, or showed a relative standard deviation > 30%, or had a similarity value < 200 were excluded from analysis. Supervised orthogonal partial least-squares discrimination analysis (OPLS-DA) was conducted using metaX to discriminate the different variables between two groups [34]. KEGG functional annotation and enrichment analysis were performed using MetaboAnalyst 5.0 (https://www.metaboanalyst.ca/), and the pathways with greater impact values and lower *P* values were regarded as the key pathways [35].

### Microbial co-occurrence network analysis

Inter-domain ecological networks (IDENs) were constructed using a Random Matrix Theory-based method as previous described [36]. The 50 differential bacteria (> 0.01%) and 30 differential archaea (> 0.0001%) with the greater relative abundances were selected for subsequent network analysis. The co-occurrence network among these microbes were calculated by Spearman's rank correlation analysis. After threshold scanning using a Random Matrix Theory-based approach, IDENs were constructed with a cutoff 0.82 and the network structure was visualized using Cytoscape v3.7.1 [37].

### Pan-genomic analysis of selected rumen microbes

As the *Prevotella*, *Succinimonas*, *Succinatimonas*, *Alistipes*, *Bacteroides*, *Methanosphaera*, and *Methanobrevibacter* were key rumen microbial taxa significantly correlated with milk yield, a pan-genomic analysis was performed to further characterize the conserved functional features of these selected microbes. The genome sequences of these microbial strains were downloaded from the NCBI. Based on the criteria of completeness > 90% and contamination < 5%, a total of 88 *Prevotella*, 9 *Succinimonas*, 126 *Succinatimonas*, 128 *Alistipes*, 168 *Bacteroides*, 37 *Methanosphaera*, and 142 *Methanobrevibacter* were selected for subsequent analysis. Since *Succinimonas* and *Succinatimonas* are both members of the Succinivibrionaceae family and exhibit a close evolutionary relationship [38], and given the limited number of available *Succinimonas* genomes, these genomes were grouped together (under Succinivibrionaceae) for subsequent pan-genome analysis. A pan-genome calculation was performed using IPGA (https://nmdc.cn/ipga/) as described previously [39].

Briefly, the input genomes were quality controlled using checkM [40] and GUNC [41] to check for completeness and contamination. The quality-controlled genomic details of these seven genera are summarized in Supplementary Table S2. Next, genes of all filtered genomes were predicted and annotated using Diamond against the Clusters of Orthologous Groups database [42]. The set of genes shared by 95% of all the genomes in our dataset was defined as the core genes, while genes partially shared between members (accessory genes) and unique to single members (unique genes) were defined as dispensable genes. Different pan-genomic profiles were created in IPGA using 4 approaches: Roary [43], panX [44], Panaroo [45], and PPanGGoLiN [46]. Of note, PPanGGoLiN generated the highest scoring profiles and was retained for downstream analyses.

### Structural equation modeling construction

Structural equation models were constructed using the lavaan package [47] in *R* to evaluate the direct correlations between differential microbial species, differential metabolites from rumen, plasma, and milk, and ECM yield. Specifically, key elements used in the structural equation models were derived from the significantly different microbes, metabolites, and phenotypes (ECM yield) identified through our correlation analysis, network analysis, and pan-genomic analysis. The model was constructed based on the underlying logic that ruminal microbes influence lactation performance via ruminal metabolites, as noted by previous studies [48, 49]. The goodness-of-fit of the structural equation model was verified by a chi-square to degrees of freedom ratio (χ^2^/df), the root mean square error (RMSE), and the comparative fit index (CFI). The model was determined to have a good fit when χ^2^/df between 2 and 3, the RMSEA approaching 0, and CFI nearing 1 [50].

### Statistical analysis

A power analysis (using G*Power v3.1.9.7) was carried out using lactation performance as the primary response variable to obtain a power of 0.80 under the significance level of 0.05 according to similar previous studies [18, 51, 52], confirming that our sample size (*n* = 7 per group) was statistically sufficient. Differences in rumen fermentation and plasma parameters among the four groups were analyzed using a general linear model in SPSS 20.0 (SPSS INC, USA), with period (POSP vs. PREP), ECM yield (high vs. low), and the period × ECM interaction set as fixed factors. The variance for each cow was used as the random effect. Multiple group comparisons were performed using one-way ANOVAs and Tukey's HSD post hoc test. The level of statistical significance was set at *P* < 0.05 and a tendency for significance was set at 0.05 ≤ *P* < 0.10.

For the metagenomic data, differential microbial taxa among groups were analyzed using linear discriminant analysis (LDA) effect size (LEfSe) analysis, with the significance threshold set as LDA score > 2.5. Additionally, for differential microbial taxa identified through LEfSe analysis, Wilcoxon rank-sum tests were performed to assess differences between POSP_H and POSP_L groups, as well as between PREP_H and PREP_L groups. For the metabolomic data, the variable important for the projection (VIP) value was calculated, and VIP cut-off value > 1.0 with false discovery rate (FDR) corrected* P*-value (*q* value) < 0.05 (Student's t-test) were used to identify different metabolites. The significantly different genes encoding enzymes from metagenomic data and significantly different metabolites among four groups were determined using the Kruskal–Wallis H test. Comparisons between means were undertaken using Tukey–Kramer's test when the group effect was significant. Among these significantly different enzymes, only those directly involved in energy metabolism, as determined by KEGG, was used in the pathway analysis. Correlation analysis among the ruminal microbiota, rumen fermentation parameters, plasma metabolites, and ECM yield were tested by Spearman's rank correlation. Correlation analysis between bacterial species, metagenomic genes, or metabolites were also tested by Spearman's rank correlation. These correlations were visualized using Cytoscape v3.7.1 [37]. All statistical significance thresholds of our omics related data were set at *q* < 0.05.

## Results

### Differences in rumen fermentation and plasma parameters between high and low yield cows during the transition period

**Fig. 1:**
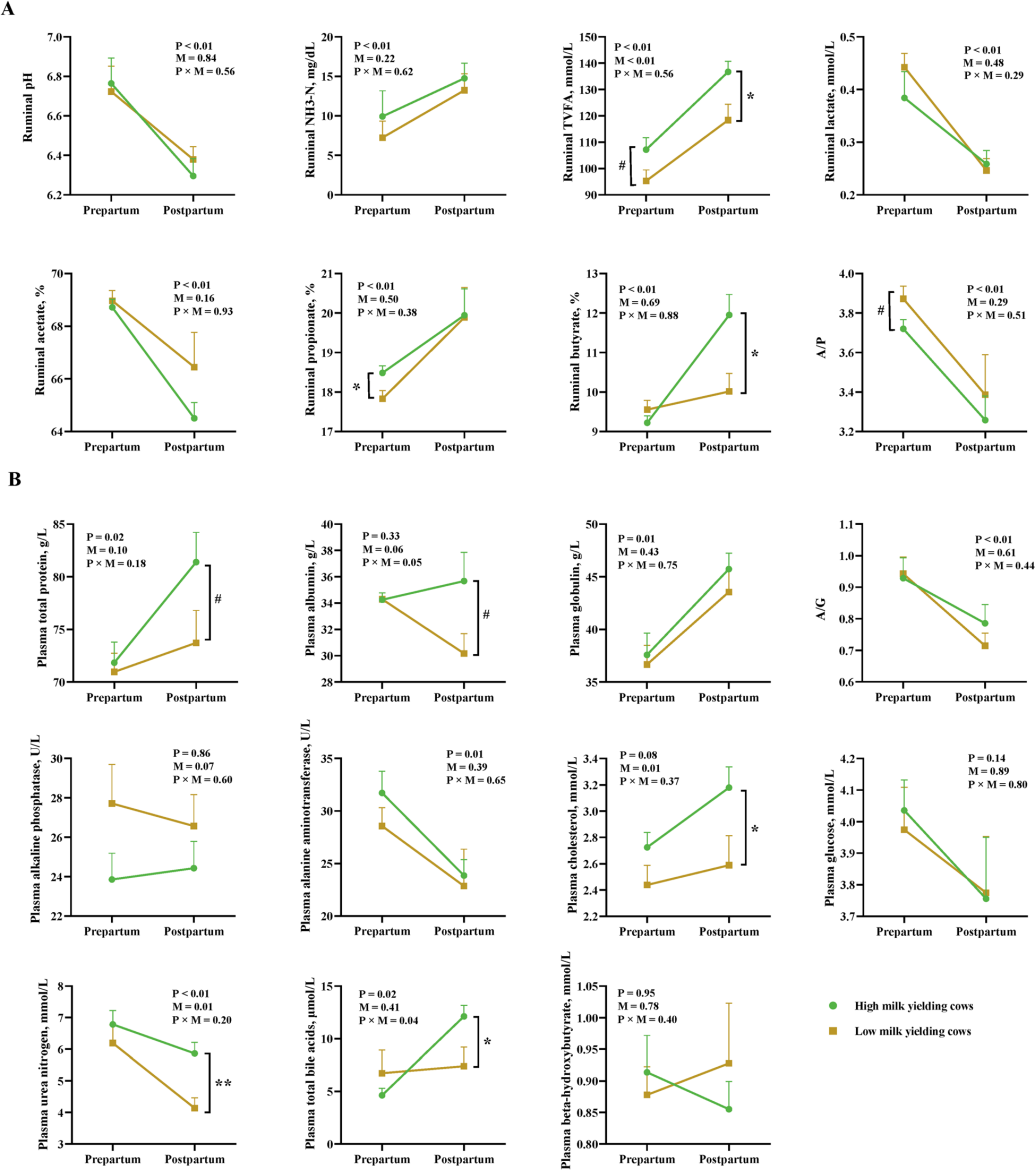
Differences in ruminal fermentation (**A**) and plasma (**B**) parameters between high and low yield dairy cows during the transition period (*n* = 7 per group). The data were analyzed using a general linear model. The error bars indicate standard error of means. Statistical comparisons between POSP_H vs POSP_L and PREP_H vs PREP_L were conducted using a Wilcoxon rank-sum test, with significance levels indicated by * *P* < 0.05, ** *P* < 0.01, and # *P* < 0.1. ECM: energy-corrected milk; NH_3_-N: ammonia nitrogen; TVFA: total volatile fatty acids; A/P: acetate to propionate ratio; A/G: albumin to globulin ratio; P = main effect of period; M = main effect of ECM yield; P × M = period × ECM production interaction. PREP_H: prepartum cows with high ECM postpartum; PREP_L: prepartum cows with low ECM postpartum; POSP_H: postpartum cows with high ECM; POSP_L: postpartum cows with low ECM

Total ruminal VFA concentration was influenced by both period and ECM yield (*P* < 0.01, Fig. 1A). Significant effects of period were observed in rumen pH, concentrations of NH_3_-N and lactate, proportions of acetate, propionate, and butyrate, and acetate to propionate ratio (A/P). Compared with POSP_L group, the POSP_H group had greater total VFA concentration and butyrate proportion (*P* < 0.05). Compared with PREP_L group, the PREP_H group had significantly greater propionate proportion (*P* < 0.05), with a tendency for increased total VFAs (*P* < 0.1) and a lower A/P ratio (*P* < 0.1).

Plasma total protein, globulin, and total BA concentrations, ratio of albumin to globulin (A/G), and alanine aminotransferase activity were significantly influenced by the effects of period (*P* < 0.05, Fig. 1B). Plasma cholesterol concentration was significantly influenced by ECM (FDR *P* < 0.05), with a tendency for the period effect (*P* = 0.08). Plasma urea nitrogen concentration was influenced by both period and ECM (*P* < 0.05). The interactions of period and ECM was significant for total BA concentration (*P* < 0.05). Compared with POSP_L group, the POSP_H group had greater concentrations of plasma cholesterol, urea nitrogen and total BA (*P* < 0.05), with a tendency for increased total protein and albumin concentration (*P* < 0.1). Based on Spearman's correlation analysis, the ruminal concentration of total VFAs and the proportion of butyrate were found to be positively associated with milk yield (*r* > 0.60, *q* < 0.05, Fig. S2). The concentrations of total protein and BUN in plasma were positively associated with milk and ECM yield (*r* > 0.50, *q* < 0.05).

### Differences in metabolomics profiling between high and low yield cows during the transition period

**Fig. 2:**
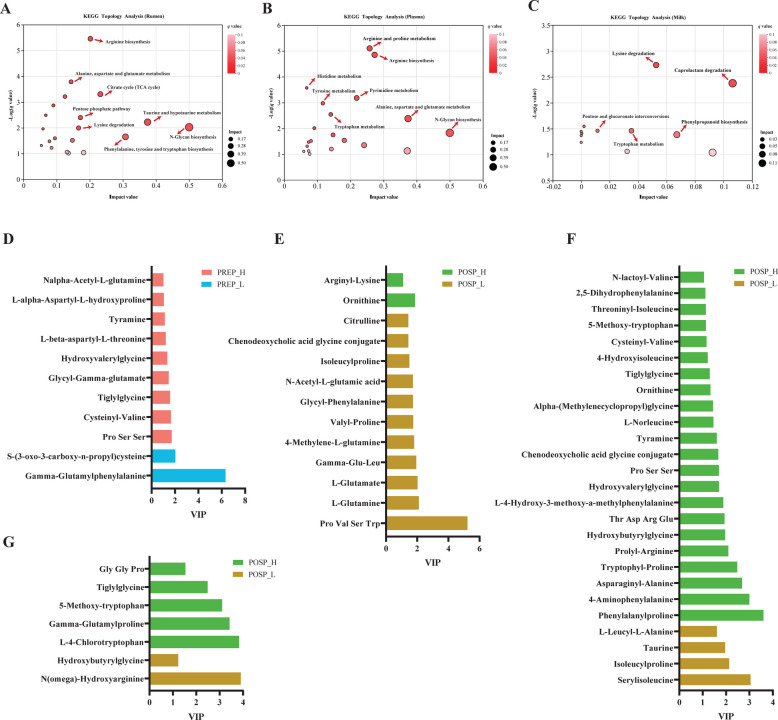
Metabolomic profiles of the ruminal, plasma, and milk samples of high and low yield dairy cows during the transition period (*n* = 7 per group). **A** Ruminal metabolomics pathway analysis. A total of 537 differential metabolites in the rumen from four comparisons (PREP_H vs. PREP_L, POSP_H vs. POSP_L, POSP_H vs. PREP_H, and POSP_L vs. PREP_L) were selected. **B** Plasma metabolomics pathway analysis. A total of 617 differential metabolites in the plasma from four comparisons (PREP_H vs. PREP_L, POSP_H vs. POSP_L, POSP_H vs. PREP_H, and POSP_L vs. PREP_L) were selected. **C** Milk metabolomics pathway analysis using the 93 significantly different metabolites between POSP_H and POSP_L group. **D** Differential amino acid related metabolites in plasma metabolome between PREP_H and PREP_L group. **E** Differential amino acid related metabolites in ruminal metabolome between POSP_H and POSP_L group. **F** Differential amino acid related metabolites in plasma metabolome between POSP_H and POSP_L group. **G** Differential amino acid related metabolites in milk metabolome between POSP_H and POSP_L group. The impact value is a quantitative metric employed in metabolic pathway analysis to assess the relative contribution of individual pathway to global metabolic alterations. VIP: Variable Importance in Projection. PREP_H: prepartum cows with high energy-corrected milk yield (ECM) postpartum; PREP_L: prepartum cows with low ECM postpartum; POSP_H: postpartum cows with high ECM; POSP_L: postpartum cows with low ECM

A total of 537 and 617 differential metabolites were determined from our four comparisons (PREP_H vs. PREP_L, POSP_H vs. POSP_L, POSP_H vs. PREP_H, and POSP_L vs. PREP_L) in the rumen and plasma, respectively, and 93 differential metabolites were determined from milk samples from POSP_H vs. POSP_L, as identified using VIP filtering (Tables S3-11). Ruminal significantly different metabolites were mapped to numerous metabolic pathways, including arginine biosynthesis; the citrate cycle (TCA cycle); alanine, aspartate and glutamate metabolism; lysine degradation; and phenylalanine, tyrosine and tryptophan biosynthesis (*q* < 0.05, Fig. 2A). Significantly different metabolites in plasma were found to map to metabolic pathways including arginine and proline metabolism; arginine biosynthesis; histidine metabolism; tyrosine metabolism; tryptophan metabolism; and alanine, aspartate and glutamate metabolism (*q* < 0.05, Fig. 2B). Significantly different metabolites in milk were mapped to lysine degradation and tryptophan metabolism (*q* < 0.05, Fig. 2C).

We found that all metabolomes, except for the rumen fluid in prepartum cows, showed differences in the relative concentrations of amino acids, peptides, and their derivatives between high-yielding and low-yielding cows (Table S3-11). The relative concentrations of 9 metabolites (including Pro-Ser-Ser, cysteinyl-valine, tiglylglycine, and tyramine) were greater in the plasma of PREP_H group, compared with the PREP_L group, whereas 2 metabolites (gamma-glutamylphenylalanine and S-(3-oxo-3-carboxy-n-propyl)-cysteine) were lower in the plasma of PREP_H group (Fig. 2D). Ruminal ornithine and arginine-related derivatives (arginyl-lysine and ornithine) were increased, but glutamate-related derivatives (including L-glutamine, L-glutamate, gamma-Glu-Leu, and 4-methylene-L-glutamine) were decreased in the POSP_H group compared with the POSP_L group (Fig. 2E). The relative concentrations of bile acid-related derivatives (chenodeoxycholic acid glycine conjugate), arginine-related derivatives (ornithine, prolyl-Arginine, and Thr Asp Arg Glu), and tryptophan-related derivatives (tryptophyl-proline and 5-methoxy-tryptophan) were greater in the plasma of POSP_H group compared with the POSP_L group, while 4 metabolites (serylisoleucine, isoleucylproline, taurine and L-leucyl-L-alanine) were lower (Fig. 2F). Only 2 metabolites (N(omega)-hydroxyarginine and hydroxybutyrylglycine) were decreased in the milk of POSP_H cows compared with the POSP_L group (Fig. 2G). In addition, the relative concentrations of tryptophan-related derivatives (including 5-methoxy-tryptophan and L-4-chlorotryptophan) were greater in the milk of the POSP_H group than in the POSP_L group (Fig. 2G).

### Differences in rumen microbial community between high and low yield cows during the transition period

**Fig. 3:**
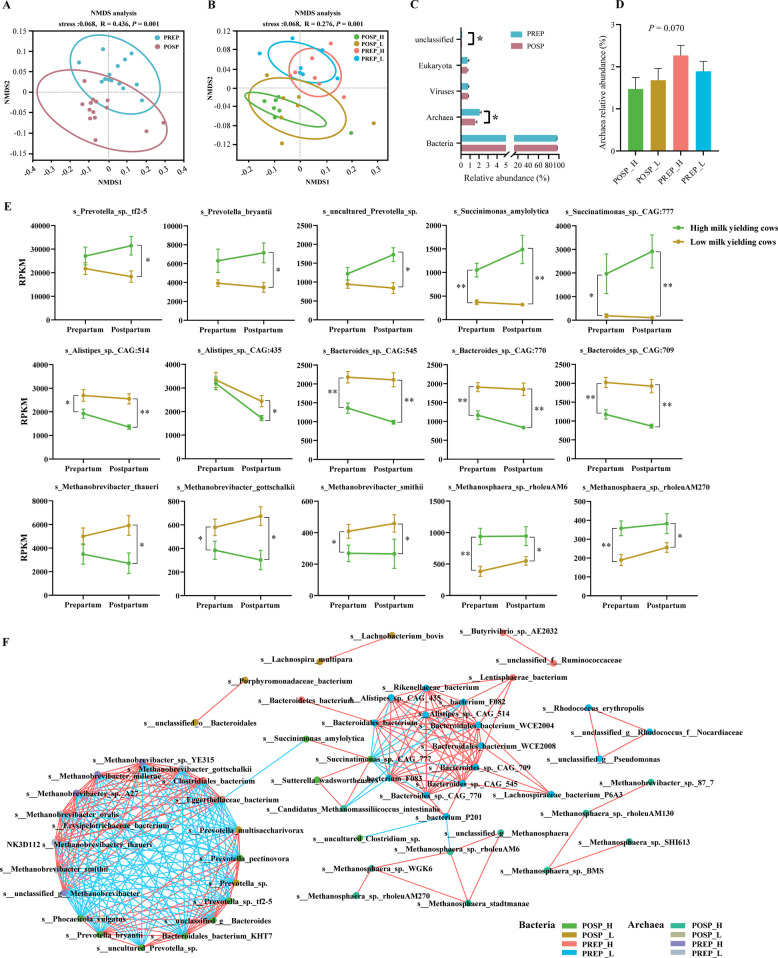
Differences in ruminal microbial structure related to period and postpartum ECM in transition cows. **A** Non-metric multidimensional scaling analysis (NMDS) of the ruminal microbiomes between PREP and POSP groups (*n* = 14). **B** NMDS analysis of ruminal microbiomes among PREP_H, PREP_L, POSP_H, and POSP_L groups (*n* = 7). **C** Comparison of microbial domains between PREP and POSP groups (*n* = 7). The statistical analysis was performed using a Wilcoxon rank-sum test, and * indicates *q* < 0.05. **D** Comparison of archaea among PREP_H, PREP_L, POSP_H, and POSP_L groups (*n* = 7). The statistical analysis was performed using the Kruskal–Wallis H test. **E** Differences in selected ruminal species between high and low yield dairy cows during the transition period (*n* = 7). In Wilcoxon rank-sum test, * indicates *q* < 0.05 and ** indicates *q* < 0.01. RPKM: reads per kilobase per million mapped reads. **F** Visualization of microbial interactions in IDENs incorporating ruminal bacterial and archaeal species (*n* = 28). Correlations were assessed using Spearman's correlation and all correlations with a *q* value < 0.05 were considered statistically significant. Red lines indicated positive correlations, and blue lines indicate negative correlations. The colors of nodes indicate the group to which the species belongs. All graphical data are displayed as mean ± SEM. PREP_H: prepartum cows with high energy-corrected milk yield (ECM) postpartum; PREP_L: prepartum cows with low ECM postpartum; POSP_H: postpartum cows with high ECM; POSP_L: postpartum cows with low ECM

Based on our NMDS and ANOSIM analysis, there were significant differences in the rumen microbial community between the PREP and POSP groups (*P* < 0.01, Fig. 3A), between the POSP_H and PREP_H groups (*P* = 0.003), and between the POSP_L and PREP_L groups (*P* < 0.01, Fig. 3B, Table S12). There was a tendency towards significance between the POSP_H and POSP_L groups (*P* < 0.1). The PREP group had a greater relative abundance of archaea than the POSP group (*q* < 0.05, Fig. 3C). There was a tendency towards significance among the PREP_H, PREP_L, POSP_H, and POSP_L groups with respect to archaeal abundance, with the PREP_H group having the greatest relative abundance (*q* < 0.1, Fig. 3D). LEfSe analysis revealed that *Prevotella* (10 species) and Succinivibrionaceae (1 *Succinimonas* species and 2 *Succinatimonas* species) exhibited the highest number of species with significantly increased relative abundances in the POSP group, whereas Bacteroidaceae (5 species) and *Alistipes* (2 species) showed the greatest species-level enrichment in the PREP group (LDA > 2.5, *q* < 0.05, Fig S3A). Among the PREP_H, PREP_L, POSP_H, and POSP_L groups, the POSP_H group had the greatest abundances of *Prevotella_sp_tf2_5*, *Prevotella_bryantii*, *uncultured_Prevotella_sp*, *Succinatimonas_sp_CAG_777* and *Succinimonas_amylolytica*, while the PREP_L group had the greatest abundances of *Bacteroides_sp_CAG_770*, *Bacteroides_sp_CAG_709*, *Bacteroides_sp_CAG_545*, *Alistipes_sp_CAG_435* and *Alistipes_sp_CAG_514* (*q* < 0.05, Fig. S3B and Fig. 3E). Compared with the PREP group, the abundances of several *Methanosphaera* species were increased, but several *Methanobrevibacter* species were decreased in the POSP group (LDA > 2.5, *q* < 0.05, Fig. S3C). Among the four groups, the POSP_H group had the greatest abundances of several *Methanosphaera* species (including *Methanosphaera_sp_rholeuAM6* and *Methanosphaera_sp_rholeuAM270*), while the PREP_L group had the greatest abundances of several *Methanobrevibacter* species (including *Methanobrevibacter_thaueri*, *Methanobrevibacter_gottschalkii*, and *Methanobrevibacter_smithii*) (*q* < 0.05, Fig. S3D and Fig. 3E).

To further investigate species interactions within the rumen microbial community, differential bacteria with relative abundances > 0.01% and differential archaea with relative abundances > 0.0001% were selected for co-occurrence network analysis. The microbial co-occurrence network comprised two major modules: one dominated by multiple *Prevotella* and *Methanobrevibacter* species, and the other featuring two Succinivibrionaceae members alongside several *Bacteroides* and *Alistipes* species (Fig. 3F). Two Succinivibrionaceae species (*Succinatimonas_sp._CAG_777* and *Succinimonas_amylolytica*), which were increased in the POSP_H group, had pivotal roles in the network, connecting the two network modules together (Fig. 3F). Several *Prevotella* species, including *Prevotella_sp_tf2_5*, *Prevotella_bryantii*, *Prevotella_pectinovora*, and an *uncultured_Prevotella_sp*, that were increased in the POSP_H group had positive correlations with each other (*r* > 0.80, *q* < 0.05). However, these *Prevotella* species were negatively correlated with some archaeal species (including *Methanobrevibacter_thaueri*, *Methanobrevibacter_gottschalkii*, and *Methanobrevibacter_smithii*) that were increased in the PREP_L group (|*r|*> 0.90, *q* < 0.05). We also found that *Succinatimonas_sp._CAG_777* had negative correlations with some bacterial species (*Alistipes_sp._CAG_514*, *Alistipes_sp._CAG_435*, *Bacteroides_sp._CAG_545*, *Bacteroides_sp._CAG_709*, and *Bacteroides_sp._CAG_770*) that were increased in the PREP_L group (|*r|*> 0.80, *q* < 0.05).

Spearman's rank correlation was used to further investigate the associations between these differential microbes and rumen fermentation parameters and plasma metabolites. Several bacterial species increased in the POSP_H group, including *Prevotella_sp._tf2-5*, *Prevotella_bryantii*, *Succinatimonas_sp._CAG_777*, *uncultured_Prevotella_sp*, and *Succinimonas_amylolytica* were positively associated with the ruminal concentrations of acetate, propionate, butyrate and total VFAs, as well as the proportions of propionate and butyrate in part or all (*r* > 0.41, *q* < 0.05, Fig. S4A). In contrast, these bacteria were negatively correlated with ruminal A/P and acetate proportion in part or all (|*r*|> 0.41, *q* < 0.05, Fig. S4A). The *uncultured_Prevotella_sp* had a positive correlation with milk yield (*r* = 0.63, *q* < 0.05, Fig. S4A). Conversely, some bacterial and archaeal species increased in the PREP_H group, including *Bacteroides_sp._CAG_545*, *Bacteroides_sp._CAG_709*, *Bacteroides_sp._CAG_770*, *Alistipes_sp._CAG_514*, *Alistipes_sp._CAG_435*, *Methanobrevibacter_millerae*, *Methanobrevibacter_sp._YE315*, and *Methanobrevibacter_thaueri*, were positively associated with ruminal pH, A/P, and acetate proportion (*r* > 0.40, *q* < 0.05, Fig. S4A and S4B). However, these bacterial and archaeal species were negatively associated with the ruminal concentrations of acetate, propionate, butyrate and total VFAs, and negatively associated with milk and ECM yield in part or all (*r* < −0.40, *q* < 0.05, Fig. S4A and S4B).

### The integrated metabolic pathway analysis the metagenome and metabolomes

**Fig. 4:**
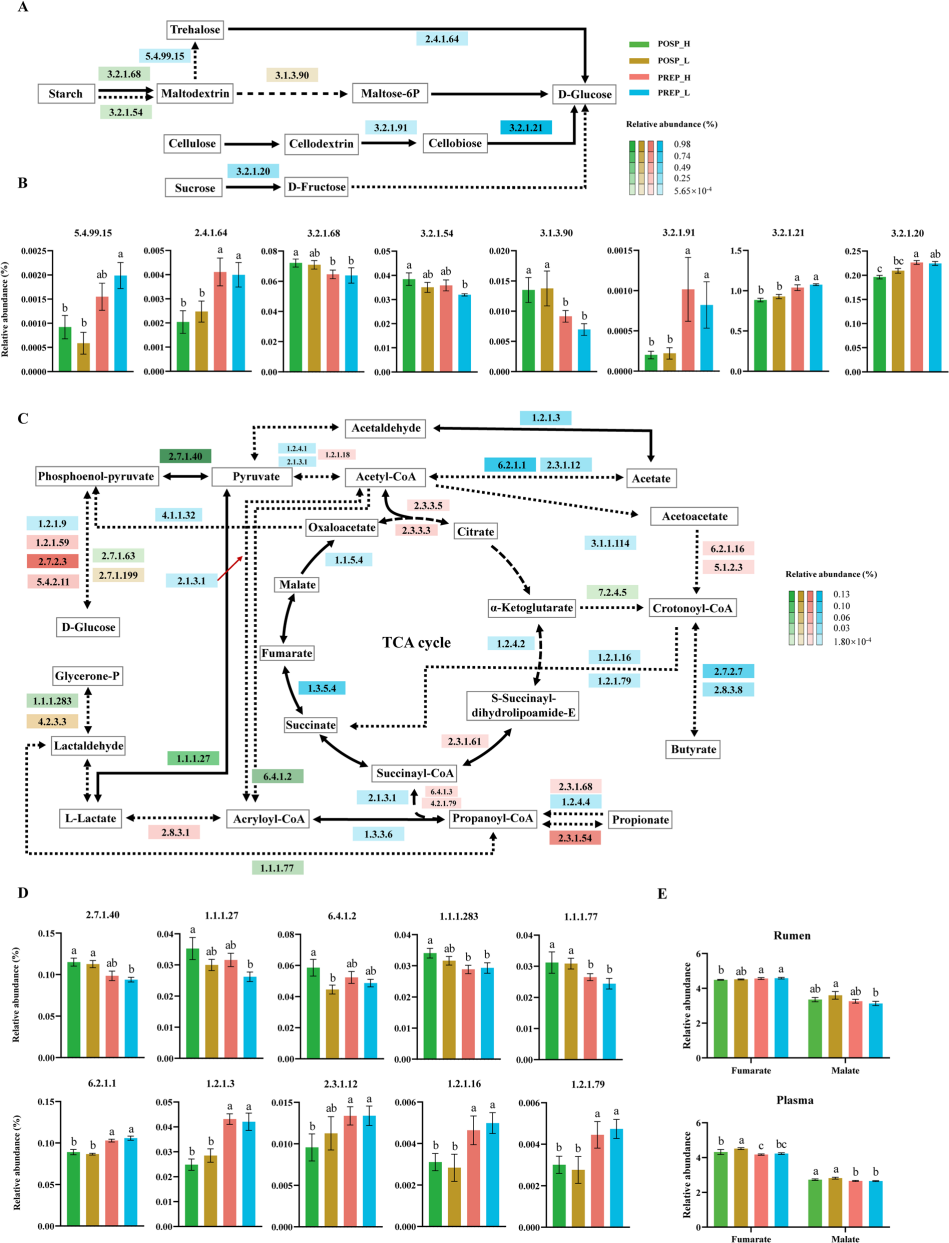
Ruminal microbial functions involved in carbohydrate and energy metabolism (*n* = 7 per group). **A** Starch and sucrose metabolism pathways. **B** Selected differential genes encoding ruminal enzymes involved in starch and sucrose metabolism pathways. **C** VFA biosynthesis pathways. **D** Selected differential genes encoding ruminal enzymes involved in VFA biosynthesis pathways. **E** Selected differential metabolites in rumen and plasma involved in the TCA cycle. The colors indicate the group in which the genes belong. Solid arrows indicate that there are no intermediate steps in the pathway. Dashed arrows indicate that an intermediate step is included in the pathway. PREP_H: prepartum cows with high energy-corrected milk yield (ECM) postpartum; PREP_L: prepartum cows with low ECM postpartum; POSP_H: postpartum cows with high ECM; POSP_L: postpartum cows with low ECM. The statistical analysis was performed using a Kruskal–Wallis H test, followed by Tukey-Kramer's post-hoc test. The different letters above bars indicate statistically significant differences (*q* < 0.05)

The metagenomic and metabolomic data were integrated to conduct metabolic pathway analysis, aiming to uncover differences in carbohydrate metabolism and amino acid metabolism among the four groups. For starch and sucrose metabolism, the cyclomaltodextrinase (EC 3.2.1.54) and isoamylase (EC 3.2.1.68), which convert starch to maltose, were greater in the POSP_H group (*q* < 0.05, Fig. 4A and B). For cellulose metabolism, the 1,4-beta-cellobiosidase (EC 3.2.1.91), maltase-glucoamylase (EC 3.2.1.20) and beta-glucosidase (EC 3.2.1.21), which are involved in the hydrolysis of cellulose and sucrose, were greater in the PREP_L group (*q* < 0.05, Fig. 4A and B). When considering the generation of short chain fatty acids and the TCA cycle, we observed that the relative abundances of genes encoding for enzymes involved in the conversion of acetate, propionate, and butyrate were significantly greater in the PREP_L group (*q* < 0.05, Fig. 4C). These include aldehyde dehydrogenase (EC 1.2.1.3), which catalyzes the inter-conversion between acetaldehyde and acetate; acetate:CoA ligase (EC 6.2.1.1) and dihydrolipoyllysine-residue acetyltransferase (EC 2.3.1.12), which catalyze the interconversion between acetyl-CoA and acetate; and butyrate kinase (EC 2.7.2.7) and acetate CoA-transferase (EC 2.8.3.8), which mediate the inter-conversion between crotonoyl-CoA and butyrate (*q* < 0.05, Fig. 4C). Of note, genes encoding for succinate-semialdehyde dehydrogenase (EC 1.2.1.16 and EC 1.2.1.79), which showed greater relative abundance in the PREP_L group, irreversibly metabolizes crotonoyl-CoA to succinate (*q* < 0.05, Fig. 4C and D). Significantly elevated abundances were also observed in POSP_H for genes encoding EC 2.7.1.40 (conversion between phosphoenolpyruvate and pyruvate), EC 1.1.1.27 (conversion between pyruvate and lactate), EC 6.4.1.2 (one of the irreversible steps in the conversion of acetyl-CoA to acryloyl-CoA), EC 1.1.1.283 (conversion between glycerone phosphate and lactaldehyde), and EC 1.1.1.77 (conversion between lactaldehyde and propanoyl-CoA) (*q* < 0.05, Fig. 4C and D). The fumarate concentration in rumen fluid was significantly lower in the POSP_H group compared with both PREP_H and PREP_L groups, and its concentration in plasma was significantly greater in POSP_H group than in PREP_H group (*q* < 0.05, Fig. 4E).

**Fig. 5:**
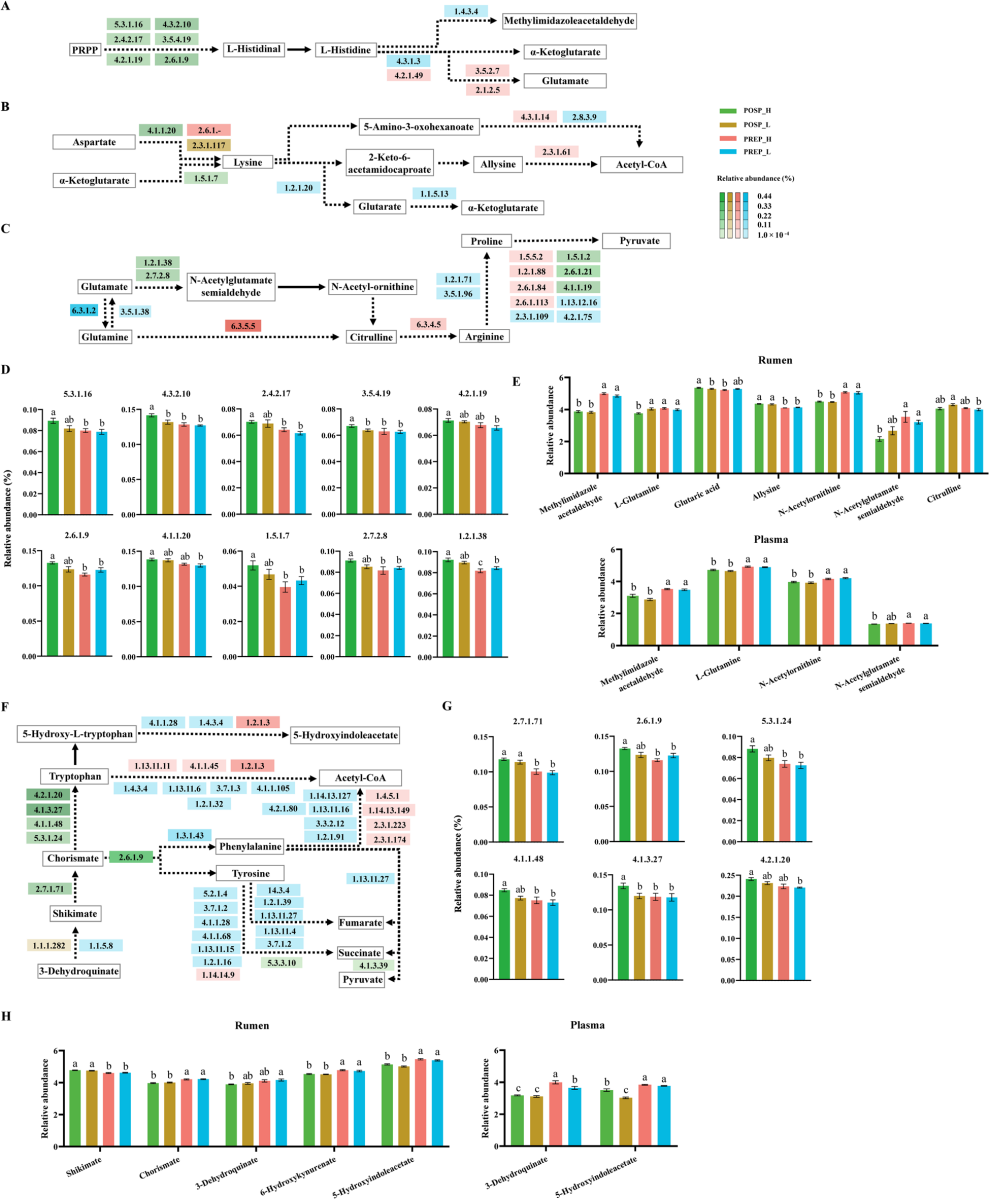
Ruminal microbial functions, ruminal metabolites, and plasma metabolites involved in amino acid synthesis and metabolism (*n* = 7 per group). **A** Histidine metabolism pathway. **B** Lysine biosynthesis and degradation pathway. **C** Arginine biosynthesis and arginine and proline metabolism pathway. **D** Selected differential genes encoding ruminal enzymes involved in histidine metabolism, lysine biosynthesis and degradation, arginine biosynthesis and arginine and proline metabolism. **E** Selected differential metabolites in the rumen and plasma involved in histidine metabolism, lysine biosynthesis and degradation, and arginine biosynthesis and arginine and proline metabolism. **F** Aromatic amino acids biosynthesis and metabolism pathway. **G** Selected differential genes encoding ruminal enzymes involved in aromatic amino acid biosynthesis and metabolism. **H** Selected differential metabolites in the rumen and plasma involved in aromatic amino acid biosynthesis and metabolism. The colors indicate the group to which the genes belong. Solid arrows indicate that there are no intermediate steps in the pathway. Dashed arrows indicate that an intermediate step is included in the pathway. PREP_H: prepartum cows with high energy-corrected milk yield (ECM) postpartum; PREP_L: prepartum cows with low ECM postpartum; POSP_H: postpartum cows with high ECM; POSP_L: postpartum cows with low ECM. The statistical analysis was performed using a Kruskal–Wallis H test, followed by Tukey-Kramer's post-hoc test. The different letters above bars indicate statistically significant differences (*q* < 0.05)

For amino acid metabolism, The POSP_H group exhibited significantly greater abundances of genes involved in histidine (6 ECs), lysine (2 ECs), arginine (2 ECs) and aromatic amino acids (6 ECs) biosynthetic pathways, compared with the other groups (*q* < 0.05, Fig. 5). Conversely, PREP_L group had significantly greater abundances of genes involved in histidine (2 ECs), lysine (3 ECs), arginine (5 ECs) and aromatic amino acid (26 ECs) degradation pathways, compared with other groups (*q* < 0.05, Fig. 5). Genes encoding for monoamine oxidase (EC 1.4.3.4, irreversibly converting histamine to methylimidazole acetaldehyde) and histidine ammonia-lyase (EC 4.3.1.3, the first irreversible step converting histamine to L-glutamate) showed significantly greater abundance in the PREP_L group (*q* < 0.05, Fig. 5A). In the arginine biosynthesis pathway, we also found two genes responsible for converting glutamate to downstream metabolites (N-acetyl-glutamate semialdehyde and N-acetylornithine), which showed significantly greater relative abundances in the POSP_H group (*q* < 0.05, Fig. 5C). Correspondingly, we observed significantly lower concentrations of both methylimidazole acetaldehyde and glutamate in the POSP_H group across plasma and rumen fluid compartments, despite concurrent reductions in N-acetyl-glutamate semialdehyde and N-acetylornithine levels (*q* < 0.05, Fig. 5E). Within the biosynthetic pathways of three aromatic amino acids (phenylalanine, tyrosine, and tryptophan), we observed significantly greater relative abundances of genes encoding for the shikimate-to-chorismate conversion (EC 2.7.1.71) and chorismate-to-tryptophan conversion (EC 5.3.1.24, EC 4.1.1.48, EC 4.1.3.27, and EC 4.2.1.20) in the POSP_H group, whereas genes encoding enzymes responsible for 5-hydroxy-tryptophan to 5-hydroxyindoleacetate conversion (EC 4.1.1.28 and EC 1.4.3.4) showed significantly elevated abundances in the PREP_L group (*q* < 0.05, Fig. 5F). In the rumen fluid metabolome, shikimate abundance was significantly greater in the POSP_H group compared with both PREP_H and PREP_L groups, while chorismate and 5-hydroxyindoleacetate showed significantly lower relative abundances (*q* < 0.05, Fig. 5H). Similarly, plasma metabolome analysis revealed significantly reduced 5-hydroxyindoleacetate levels in POSP_H group relative to PREP_H and PREP_L groups (*q* < 0.05, Fig. 5H). To further elucidate the associations of differential taxa with metagenomic functional genes and metabolites, the bacterial species increased in our POSP_H group, which included three *Prevotella* species (*Prevotella_sp_tf2_5*, *Prevotella_bryantii*, and *uncultured_Prevotella_sp*) and two Succinivibrionaceae species (*Succinatimonas_sp._CAG_777* and *Succinimonas_amylolytica*), were further selected. These five species had multiple negative correlations with microbial genes encoding for enzymes involved in cellulose hydrolysis, acetogenesis, and several animo acids metabolism, but had positive correlations with genes encoding enzymes involved in starch hydrolysis and animo acids synthesis (|*r*|> 0.40, *q* < 0.05, Fig. S5).

### Pan-genomic analyses of key rumen microbes in high and low yield cows during the transition period

**Fig. 6:**
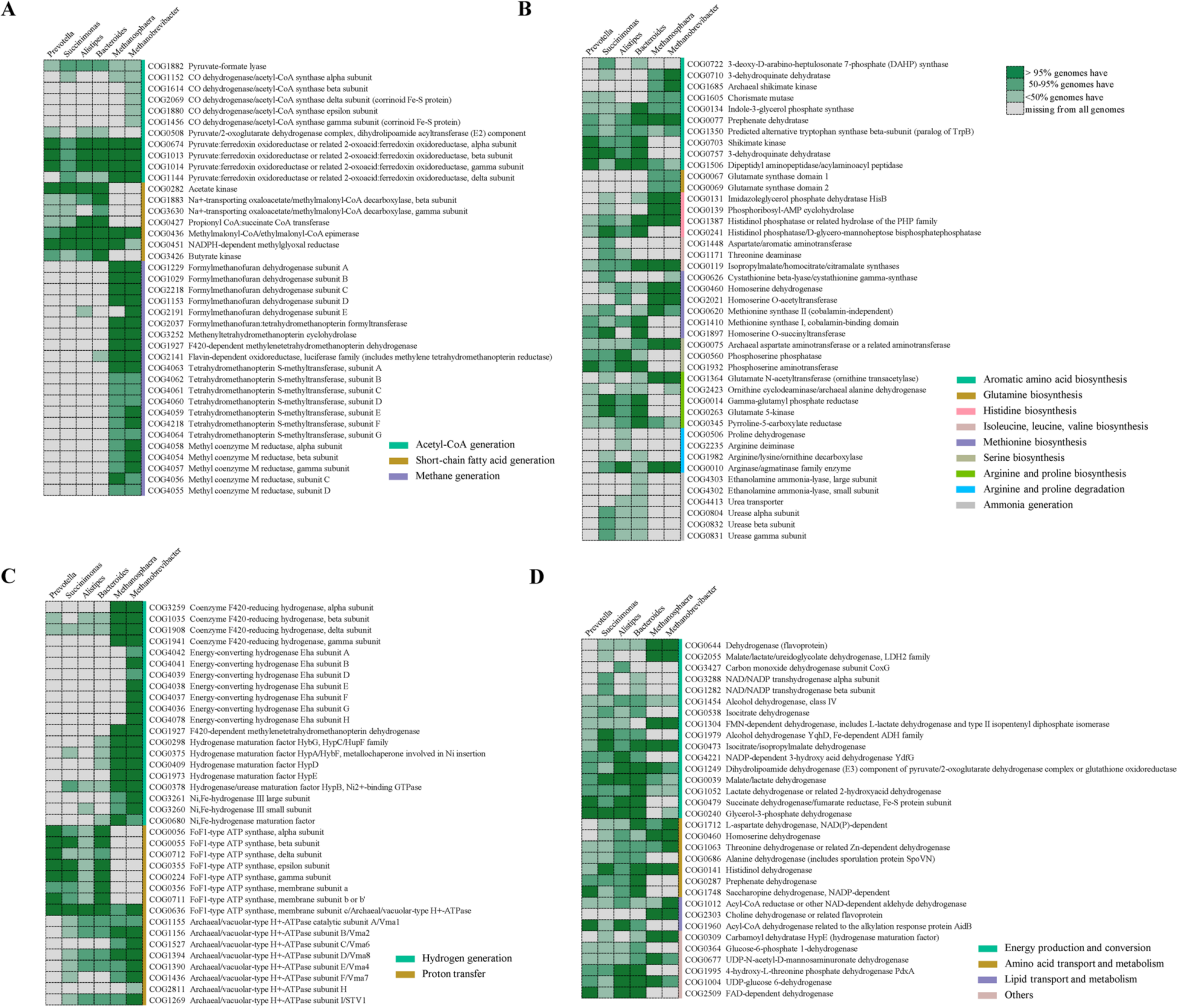
The predicted core genes of *Prevotella, Succinimonas*, *Succinatimonas, Alistipes*, *Bacteroides*, *Methanosphaera*, and *Methanobrevibacter*. **A** Genes involved in volatile fatty acid production and methanogenesis. **B** Genes involved in amino acid transport and metabolism. **C** Genes encoding hydrogenase and ATP synthases. **D** Genes encoding dehydrogenase. The colors of cells display the proportion of genes in each genus

As *Prevotella*, *Succinimonas*, *Succinatimonas*, and *Methanosphaera* were significantly greater in the POSP_H group, and *Alistipes*, *Bacteroides* and *Methanobrevibacter* were significantly greater in the PREP_L group, these microbes were further considered as two combined clusters for the following pan-genomic analysis. Although the pan-genomes of these seven genera had open state, the size of the core genomes rapidly decreased with the addition of new genomes, indicating that the core genomes are closed (Fig. S6). Multiple gene clusters encoding CO dehydrogenase/acetyl-CoA synthase (responsible for acetyl-CoA synthesis) were exclusively found in *Methanobrevibacter* genomes (Fig. 6A). Among the gene clusters encoding pyruvate:ferredoxin oxidoreductase or related 2-oxoacid:ferredoxin oxidoreductase (responsible for acetyl-CoA synthesis), three gene clusters (COG0674, COG1013, and COG1014) were conserved in the genomes of five microbial genera except for Succinivibrionaceae (*Succinimonas* and *Succinatimonas*), while one gene cluster (COG1144) was conserved in the genomes of *Methanosphaera* and *Methanobrevibacter* (Fig. 6A). Compared with *Prevotella* and Succinivibrionaceae (*Succinimonas* and *Succinatimonas*), the gene encoding propionyl-CoA:succinate CoA transferase (COG0427) was conserved in the genomes of *Alistipes* and *Bacteroides* (Fig. 6A)*.* Some gene clusters involved in the hydrogenotrophic methanogenesis pathway, including those encoding formylmethanofuran dehydrogenase (COG2191), tetrahydromethanopterin S-methyltransferase (COG4059 and COG4218), and methyl coenzyme M reductase (COG4054, COG4057, and COG4058), were conserved in *Methanobrevibacter* compared with *Methanosphaera* (Fig. 6A). Regarding the genes clusters involved in amino acids synthesis and metabolism, *Prevotella* conservatively possess three gene clusters involved in aromatic amino acid biosynthesis, including shikimate kinase (COG0703), 3-dehydroquinate dehydratase (COG0757), and Dipeptidyl aminopeptidase/acylaminoacyl peptidase (COG1506) (Fig. 6B). The gene clusters involved in arginine and proline degradation (COG0010) were conserved in *Alistipes*, *Methanosphaera*, and *Methanobrevibacter* (Fig. 6B).

Compared with *Methanosphaera*, *Methanobrevibacter* possesses more conserved gene clusters involved in hydrogen generation, including energy-converting hydrogenase (Eha) and [NiFe]-hydrogenase III (Fig. 6C). Only a few types of FoF1-type ATP synthetases presented in the *Prevotella* and Succinivibrionaceae (*Succinimonas* and *Succinatimonas*) genomes, while *Alistipes* and *Bacteroides*, as well as *Methanobrevibacter*, additionally harbored several archaeal/vacuolar-type H^+^-ATPase (Fig. 6C). Both *Prevotella* and Succinivibrionaceae (*Succinimonas* and *Succinatimonas*) contain five conserved dehydrogenase-encoding gene clusters (Fig. 6D). In contrast, *Alistipes*, *Bacteroides*, and *Methanobrevibacter* conservely contain 9, 13, and 11 of these gene clusters, respectively (Fig. 6D). There were 6 dehydrogenase-encoding gene clusters (COG0644, COG1454, COG1979, COG0473, COG1249, COG0039) associated with energy production and conversion were presented with lower proportions in *Prevotella* genomes compared with either *Alistipes* or *Bacteroides* (Fig. 6D). Two dehydrogenase gene clusters associated with amino acid transport and metabolism were present with lower proportions in both *Prevotella* and the Succinivibrionaceae (*Succinimonas* and *Succinatimonas*) genomes, when compared with either *Alistipes* or *Bacteroides* (Fig. 6D).

### Structural equation modeling reveals rumen-plasma-milk metabolic axis driving postpartum lactation performance in transition cows

**Fig. 7:**
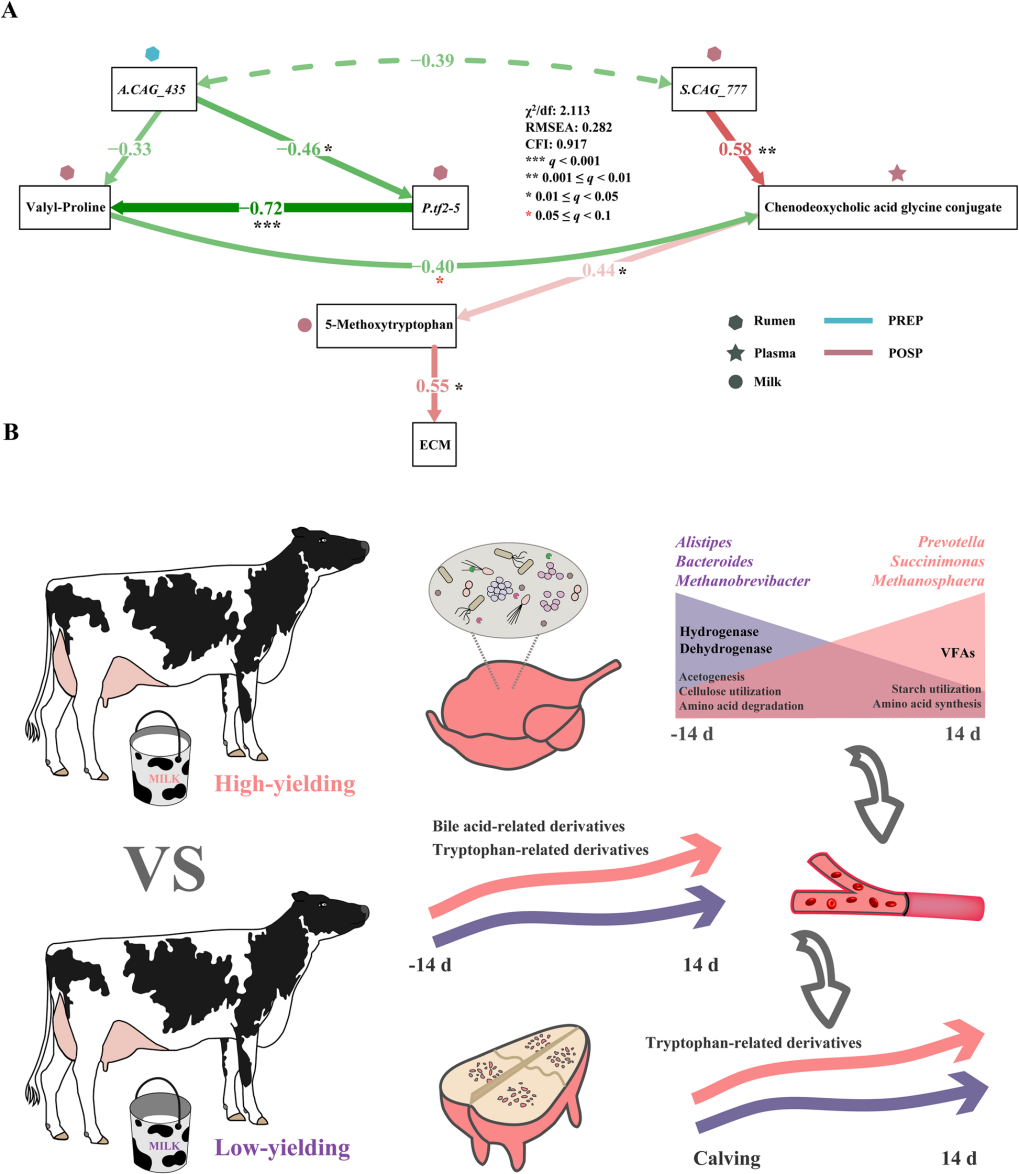
Structural equation modeling reveals rumen-plasma-milk metabolic axis driving postpartum lactation performance in transition cows. **A** Structural equation modelling with differential ruminal microbial species; differential ruminal, plasma, and milk metabolites; and energy-corrected milk yield (ECM) (*n* = 14). Hexagons, stars, and circles symbolize the locations of microbes or metabolites. Colors indicate the respective periods in which these microbes or metabolites are present. Red arrows represent positive paths and green arrows represent negative paths. The solid lines denote statistically significant relationships, while dashed lines indicate statistically non-significant ones. The line widths are proportional to the standardized path coefficients and adjacent numbers indicate the effect size of the relationship. *P.tf.2–5*: *Prevotella_sp_tf.2–5*. *S.CAG_777*: *Succinatimonas_sp_CAG_777*. *A.CAG_435*: *Alistipes_sp_CAG_435*. ECM, energy-corrected milk. Significance levels are as follows: * (red) indicates *q* < 0.1, * (black) indicates *q* < 0.05, ** indicates *q* < 0.01, and *** indicates *q* < 0.001. χ^2^/df: chi-square to degrees of freedom ratio; RMSEA, root mean square error of approximation; CFI, comparative fit index. **B** Schematic diagram illustrating how transitional rumen microbial dynamics associated blood metabolism and milk metabolism in high- and low-yield dairy cows. Low-yield dairy cows (purple) exhibited higher relative abundances of *Alistipes*, *Bacteroides*, and *Methanobrevibacter* in the rumen during the transition period, coinciding with increased frequency of genes encoding hydrogenase and dehydrogenase. In contrast, high-yield cows (pink) showed enrichment of *Prevotella*, *Succinimonas*, and *Methanosphaera*, which are taxa associated with volatile fatty acid (VFA) metabolism and amino acid synthesis pathways. These ruminal microbiota variations may increase bile acid-related derivatives in circulation and tryptophan-related derivatives in both circulation and milk, consequently improving milk production in dairy cattle

Finally, a structural equation model that incorporated our ruminal metagenomics, ruminal metabolomics, plasma metabolomics, and milk metabolomics data was constructed to explore the associations of ruminal microbes with postpartum ECM. This structural equation model was established by the logic of physiological metabolism processes from rumen to plasma and then to milk yield and composition (RMSE = 0.282, CFI = 0.917, Fig. 7A). We found that *Alistipes_sp_CAG_435* in the rumen of prepartum cows was negatively correlated with *Prevotella_sp_tf2_5* in the rumen of postpartum cows (*r* = −0.46, *q* < 0.05, Fig. 7A). Furthermore, we found that *Prevotella_sp_tf2_5* in the rumen of postpartum cows was negatively correlated with ruminal valyl-proline, while *Succinatimonas_sp._CAG_777* showed a strong positive correlation with plasma chenodeoxycholic acid glycine conjugate (|*r|*> 0.58, *q* < 0.01, Fig. 7A). The plasma chenodeoxycholic acid glycine conjugate was further significantly correlated with the milk 5-methoxytryptophan, which in turn exhibited a positive correlation with ECM yield in postpartum dairy cows (*r *= 0.55, *q* < 0.05, Fig. 7A).

## Discussion

A highly dynamic rumen microbial community appears to be an important characteristic of ruminants during the transition period [9–11]. By integrating ruminal metagenomics, ruminal metabolomics, plasma metabolomics, and milk metabolomics, we investigated the relationship between transitional ruminal microbes and milk production in dairy cows. Specifically, greater relative abundances of several species of *Prevotella*, *Succinimonas*, *Succinatimonas*, and *Methanosphaera* were observed in postpartum dairy cows, particularly those with high milk yield. Conversely, lower relative abundances were noted in bacteria belonging to *Alistipes* and *Bacteroides*, as well as the archaea *Methanobrevibacter*. As one of the most abundant microbes in the rumen of adult dairy cows, *Prevotella* has been reported to be positively associated with milk fat concentrations and milk protein yields [18, 53]. Succinivibrionaceae, parcticularly *Succinimonas_amylolytica*, are amylolytic, amylodextrin- and maltose-utilizing bacteria, which can grow rapidly in high-starch environments, thereby promoting ruminal VFA production and lowering pH [18, 54]. Among increased microbes in prepartum cows, *Alistipes* and *Bacteroides* have been reported to have a wide range of polysaccharide-degrading activities, and their abundances decreased with the reduced fiber content in their diets [54, 55]. Notably, *Alistipes* can only produce succinate and acetate as its main metabolic end products [56] and contribute minimally to propionate production when cows are fed a high-starch diet. A meta-analysis of bovine gastrointestinal tract microbiota revealed that the relative abundance of *Alistipes* was much greater in the feces than in the rumen [57], which suggests that it is more advantageous in a nutrient-poor environment for *Alistipes*. Our structural equation model demonstrated that *Prevotella_sp_tf.2–5* and *Succinatimonas_sp_CAG-777*, along with plasma chenodeoxycholic acid glycine conjugate and milk 5-methoxytryptophan, were positively correlated with ECM yield in postpartum dairy cows. In contrast, certain prepartum ruminal microbes (e.g., *Alistipes_sp_CAG-435*) exhibited negative associations with ECM yield. Thus, *Prevotella, Succinimonas,* and *Succinatimonas* might play a pivotal role in promoting milk production in dairy cows.

Previous reports have also suggested that the presence of *Prevotella* is associated with lower feed efficiency [58, 59] or growth performance [60]. In contrast to other studies [18, 49, 61] where *Prevotella* played a beneficial role, some of these studies focused on beef cattle [58] and dairy goats [60]. Compared with dairy cows, dairy goats and beef cattle are typically fed diets with greater concentrate proportions, which may result in different ruminal environments from those of dairy cows. Furthermore, in a study of transition dairy cows [59], a low relative abundance of *Prevotella* was reported. One theory posits that microbial functional redundancy enables different species to perform analogous ecological functions [62], and thus *Prevotella*'s ecological niche may be occupied by other microbes under these conditions. Consequently, an enhanced focus on functional characterization is needed to explain the observed microbial taxonomic differences and their mechanistic links to lactation efficiency in dairy cows.

Our metagenomic functional analysis revealed that the relative abundances of several enzyme genes involved in acetogenesis, the TCA cycle, and the assimilation of other volatile fatty acids were significantly greater in the PREP_L group. For example, Crotonoyl-CoA (an intermediate metabolite in butanoate metabolism) can be irreversibly metabolized to succinate, with the final step catalyzed by succinate-semialdehyde dehydrogenase (EC 1.2.1.16 and EC 1.2.1.79); genes encoding these enzymes were found in greater relative abundance in the PREP_L group. Moreover, the relative abundances of TCA cycle-related genes, including EC 1.2.4.2, EC 1.3.5.4, and EC 1.1.5.4, were significantly greater in the PREP_L group. The TCA cycle is recognized as the metabolic hub connecting carbohydrate, lipid, and protein metabolism [63], which may drive the conversion of propionate and butyrate to acetate in the PREP_L group. Compared with propionate and butyrate, acetate has long been recognized as a metabolite with greater hydrogen-producing capacity, lower energy utilization efficiency, and close coupling to methanogenesis [64].

It was previously assumed that *Methanobrevibacter* are the predominant methanogens in the rumen, followed by a lower percentage of *Methanosphaera* [65]. *Methanobrevibacter* can utilize CO_2_/H_2_ for methanogenic growth via the hydrogenotrophic pathway, while *Methanosphaera* has evolved the ability to use only methanol [66]. In addition, the affinity and thresholds for H_2_ are lower for *Methanosphaera* compared with *Methanobrevibacter* [66]. The reversible interconversion of molecular hydrogen and protons is one of the most ancient metabolic reactions within microbes and plays a crucial role in metabolic homeostasis and energy conservation. Our pan-genomic analysis suggested that several genes involved in hydrogen generation were conserved in *Methanobrevibacter*, including the energy-converting hydrogenase *Eha* [67], the [NiFe]-hydrogenase III [68], and the coenzyme F420-reducing hydrogenase [69]. These results might indicate that the hydrogenotrophic pathway mediated by *Methanobrevibacter* was a major contributor to methanogenesis in prepartum or in low-yield cows. Some studies have shown that *Bacteroides* can be synergistically symbiotic with *Methanobrevibacter* by providing hydrogen or other nutrients [70, 71]. In this study, *Prevotella*, *Succinimonas*, *Succinatimonas,* and *Methanosphaera* harbored several types of FoF1-type ATP synthase, while *Alistipes* and *Bacteroides* as well as *Methanobrevibacter* additionally contain archaeal/vacuolar-type H^+^-ATPase. This suggested that the genomes of *Alistipes* and *Bacteroides* might have undergone significant gene incorporation from archaea. These results implied that the *Alistipes* and *Bacteroides* might have a functional synergistic symbiosis with *Methanobrevibacter*, potentially leading to reduced VFAs production and increased methane production in the rumen of low-yield dairy cows. In contrast, the gene clusters for hydrogenases and dehydrogenases are not conserved in the genomes of *Prevotella*, *Succinimonas*, and *Succinatimonas*. Taken together, greater relative abundance of *Prevotella*, *Succinimonas*, and *Succinatimonas* in postpartum dairy cows may indicate reduced methanogenesis and improved energy utilization efficiency.

The metagenomics functional-level results further revealed that microbial genes involved in several essential amino acids synthesis, including histidine metabolism, lysine biosynthesis, arginine biosynthesis, and phenylalanine, tyrosine and tryptophan biosynthesis, were increased in postpartum cows, especially in high-yield animals. The amino acids absorbed in vivo often have different metabolic fates. In addition to direct incorporation into milk protein, some absorbed amino acids act as energy sources in the liver or as carbon precursors of other amino acids synthesized in mammary gland cells and hepatocytes [72]. Ornithine, which was found to be increased in postpartum high-yield cows both in the rumen and plasma, can serve as a nitrogen precursor in these synthetic processes [73]. Essential AAs (EAAs) were categorized into different groups: Group 1 EAAs (histidine, phenylalanine, tyrosine, and tryptophan) are highly utilized in the liver, and their secretion in milk protein are proportional to the extraction by the mammary gland [74, 75]. In both plasma and milk metabolome, our results show that the relative concentrations of tryptophan-related derivatives were increased in high-yield postpartum cows, relative to low-yield postpartum cows, which might suggest that an increased supply of tryptophan is beneficial for milk production in postpartum dairy cows [76]. In contrast, Group 2 EAAs (including arginine and lysine) are extracted from the mammary gland in greater proportions than are secreted in milk protein, but very little extraction of Group 2 EAAs occurs in the liver [74]. Arginine is one of the essential amino acids critical for milk fat and protein synthesis in lactating dairy cows [77, 78]. This suggests that the greater supply of derivatives of arginine (arginyl-lysine and Thr-Asp-Arg-Glu) in the rumen would be advantageous for mammary gland development and recovery in high-yield postpartum cows. Furthermore, the pan-genomic analysis revealed that *Alistipes* and *Methanobrevibacter* exhibiting genetic conservation in arginine and proline degradation pathways, which might contribute to arginine depletion in the rumen. However, the genome of *Prevotella*, *Succinimonas*, and *Succinatimonas* do not have these conserved genes, which might be more conducive to facilitate host acquisition of lactation-beneficial amino acids.

It was important to note that elevated chenodeoxycholic acid (CDCA) glycine conjugate in postpartum plasma was positively associated with greater ECM. CDCA is a primary BA synthesized in the liver, and is usually found conjugated with glycine or taurine prior to secretion [79]. CDCA is highly hydrophobic and has the strongest activation of the farnesoid X receptor among the known BAs, which makes it an important signaling molecule to regulate lipid, glucose, and energy metabolism [80]. Although the regulatory mechanisms by which CDCA has been studied primarily in rodents, swine, and humans, it has not been explored in ruminants, especially in transition dairy cows. Recently, one study from our group reported interactions among bile acids, the gut microbiome, and glucose and lipid metabolism status in transition dairy cows and found a similar phenomenon [81]. Another study highlighted the potential benefits of bile acids supplementation in improving milk yields and quality, as well as influencing metabolic pathways in transition dairy cows [82]. Given the dramatic metabolic stress experienced by cows during the transition period, elevated CDCA glycine combined with other BAs in plasma might be favorable.

To our knowledge, this study is one of the first to investigate the relationship between the transition-period ruminal microbiota and divergent postpartum milk yield in dairy cows using a multi-omics analysis. Although our study sought to provide comprehensive insights, we acknowledge that there are limitations that require future work. First, numerous complex correlations among ruminal microbes were identified in this study and further in-depth investigation using approaches such as co-culture experiments are needed to confirm these interactions. Second, milk yield is not only known to be regulated by ruminal nutritional supply, but through other organs such as the liver and mammary gland. Future study should consider the rumen-hepatic and rumen-mammary gland axes to gain a more comprehensive understanding of lactation regulation.

## Conclusion

This study shows that the dynamics of the ruminal microbiome during the transition period is tightly related to milk production (Fig. 7B). High-yield postpartum cows had greater relative abundances of several *Prevotella*, *Succinimonas*, and *Succinatimonas* species which were characterized by microbial genes and metabolites involved in starch utilization and amino acid synthesis. Meanwhile, *Prevotella*, *Succinimonas*, and *Succinatimonas* also had significant negative correlations with bacteria belong to *Alistipes* and *Bacteroides* and archaeal *Methanobrevibacter*. A structural equation modeling analysis revealed that a greater relative abundance of *Alistipes*_*CAG-435* in the prepartum period was negatively associated with postpartum *Prevotella* and *Succinatimonas* species, ultimately correlating with reduced postpartum milk production. Our pan-genomic analysis further confirmed that *Alistipes*, *Bacteroides*, and *Methanobrevibacter* possess genetic conservation of hydrogenase and dehydrogenase, indicating that these microbes may be involved in hydrogenotrophic methanogenesis through degradation of dietary amino acids. In conclusion, these findings provide a deeper understanding of how ruminal microbiome-dependent mechanisms contribute to early lactation performance in transition dairy cows, which will be useful for the development of effective nutritional strategies, such as the application of probiotics and prebiotics, to improve performance and efficiency in ruminants.

## Supplementary Information

Additional file 1: Fig. S1. Experimental design for this study. Healthy multiparous Chinese Holstein dairy cows (*n* = 100) with similar last 305-d milk yields, parity, body condition scores, and due date were selected. Ruminal fluid and blood samples of all cows were collected at −14 d (14 days before calving) and 14 d (14 days after calving) 2 hours after the morning feeding. Milk samples of all cows were collected at 14 d. Average milk yield at 13 d, 14 d, and 15 d was used to calculate the yield of energy corrected milk (ECM) of all cows. Based on the ECM yield of all cows at 14 d, the 7 cows with highest ECM yield (51.07 ± 7.76 kg/d, mean ± SD) and the 7 cows with the lowest ECM yield (31.19 ± 3.36 kg/d, mean ± SD) were used for the following analysis. The selected cows were compared based on their pregnancy status: prepartum (PREP, *n* = 14) and postpartum (POSP, *n* = 14), or based on a combination of ECM and pregnancy status: prepartum cows with high ECM postpartum (PREP_H, *n* = 7); prepartum cows with low ECM postpartum (PREP_L, *n* = 7); postpartum cows with high ECM (POSP_H, *n* = 7); postpartum cows with low ECM (POSP_L, *n* = 7). Fig. S2. Correlation analysis of ECM production with differential ruminal fermentation and plasma parameters (*n* = 14). In the heatmap visualization, color gradients represent correlation magnitudes, with red indicating positive correlations and blue representing negative correlations. Correlations were assessed using Spearman's correlation. * indicates *q* < 0.05, ** indicates *q* < 0.01, and *** indicates *q* < 0.001. ECM: energy-corrected milk yield; A/G: albumin to globulin ratio; ALT: alanine aminotransferase; A/P: acetate to propionate; BUN: blood urea nitrogen; NH3-N: ammonia nitrogen; TVFA: total volatile fatty acids. Fig S3. Identification of differential ruminal bacterial and archaeal species in high and low yield dairy cows during the transition period. **A** Differential bacterial species between PREP and POSP group (*n* = 14). **B** Differential bacterial species among PREP_H, PREP_L, POSP_H, and POSP_L group (*n* = 7). **C** Differential archaeal species between PREP and POSP group (*n* = 14). **D** Differential archaeal species among PREP_H, PREP_L, POSP_H, and POSP_L group (*n* = 7). Significant differences were identified by linear discriminant analysis (LDA) effect size with LDA scores > 2.5 and *q* < 0.05. ECM: energy-corrected milk yield; PREP_H: prepartum cows with high ECM postpartum; PREP_L: prepartum cows with low ECM postpartum; POSP_H: postpartum cows with high ECM; POSP_L: postpartum cows with low ECM. Fig. S4. Correlation analysis between differential ruminal microbes and fermentation parameters, plasma metabolites, and energry-corrected milk (ECM). **A** Correlation analysis of differential bacterial species with ruminal fermentation (*n* = 28), plasma parameters (*n* = 28), and ECM yield (*n* = 14). **B** Correlation analysis of differential archaeal species with ruminal fermentation (*n* = 28), plasma parameters (*n* = 28), and ECM yield (*n* = 14). In the heatmap visualization, color gradients represent correlation magnitudes, with red indicating positive correlations and blue representing negative correlations. In Spearman's correlation, * indicates *q* < 0.05, ** indicates *q* < 0.01, and *** indicates *q* < 0.001. A/G: albumin to globulin ratio; ALT: alanine aminotransferase; A/P: acetate to propionate; BUN: blood urea nitrogen; NH3-N: ammonia nitrogen; TVFA: total volatile fatty acids. PREP_H: prepartum cows with high ECM postpartum; PREP_L: prepartum cows with low ECM postpartum; POSP_H: postpartum cows with high ECM; POSP_L: postpartum cows with low ECM. Fig. S5. Visualization of microbial interactions with ruminal microbial functions and metabolites (*n* = 28). Red lines indicated positive correlations, and blue lines indicate negative correlations. The thickness of the lines represents the magnitude of the correlation coefficients. The colors of nodes indicate the group to which the species/enzyme genes/metabolites belong. Correlations were assessed using Spearman's correlation and all correlations with a *q* value < 0.05 were considered statistically significant. Fig S6. Pan-genomes and core-genomes of *Prevotella* (A), Succinivibrionaceae (*Succinimonas* and *Succinatimonas*) (B), *Methanosphaera* (C), *Alistipes* (D), *Bacteroides* (E), and *Methanobrevibacter* (F). The red line represents the pan-genome size for each strain combination, while the blue line indicates the corresponding core genome size.

Additional file 2: Table S1. Ingredients and chemical compositions of the experimental diets. ^1^ADF, acid detergent fiber; CP, crude protein; DM, dry matter; EE, ether extract; NDF, neutral detergent fiber; NEL, net energy for lactation; calculated based on National Research Council (2001). ^2^Premix provided the following per kg: Close-up: 250 g Ca, 29 g Mg, 13 mg Na, 38 g S, 42 g Cl, 375 mg Cu, 1300 mg Mn, 1100 mg Zn, 22 mg I, 5 mg Se, 11 mg Co, 500000 IU Vitamin A, 100000 IU Vitamin D, 15 mg Vitamin E; Fresh cow: 101 g Ca, 45 Mg, 47 g Na, 2 g S, 72 g Cl, 500 mg Cu, 1100 mg Mn, 2300 mg Zn, 30 mg I, 5 mg Se, 11 mg Co, 430000 IU Vitamin A, 100000 IU Vitamin D, 13 mg Vitamin E. Table S2. Assembly and accessions of genome sequences for the 88 *Prevotella* strains, 135 Succinivibrionaceae strains (9 *Succinimonas* and 126 *Succinatimonas*), 128 *Alistipes* strains, 168 *Bacteroides* strains, 37 *Methanosphaera* strains, and 142 *Methanobrevibacter* strains used in this study. Table S3. 16 differential metabolites in the rumen between PREP_H and PREP_L group (*n* = 7 per group). Data were analyzed by Student's t-test, and only metabolites with *P* < 0.05 were shown. PREP_H: prepartum dairy cows with high energy-corrected milk in postpartum; PREP_L: prepartum dairy cows with low energy-corrected milk in postpartum. Table S4. 74 differential metabolites in the rumen between POSP_H and POSP_L group (*n* = 7 per group). Data were analyzed by Student's t-test, and only metabolites with *P* < 0.05 were shown. POSP_H: postpartum dairy cows with high energy-corrected milk; POSP_L: postpartum dairy cows with low energy-corrected milk. Table S5. 373 differential metabolites in the rumen between POSP_H and PREP_H group (*n* = 7 per group). Data were analyzed by Student's t-test, and only metabolites with *P* < 0.05 were shown. POSP_H: postpartum dairy cows with high energy-corrected milk; PREP_H: prepartum dairy cows with high energy-corrected milk postpartum. Table S6. 371 differential metabolites in the rumen between POSP_L and PREP_L group (*n* = 7 per group). Data were analyzed by Student's t-test, and only metabolites with *P* < 0.05 were shown. POSP_L: postpartum dairy cows with low energy-corrected milk; PREP_L: prepartum dairy cows with low energy-corrected milk postpartum. Table S7. 66 differential metabolites in the plasma between PREP_H and PREP_L group (*n* = 7 per group). Data were analyzed by Student's t-test, and only metabolites with *P* < 0.05 were shown. PREP_H: prepartum dairy cows with high energy-corrected milk postpartum; PREP_L: prepartum dairy cows with low energy-corrected milk postpartum. Table S8. 232 differential metabolites in the plasma between POSP_H and POSP_L group (*n* = 7 per group). Data were analyzed by Student's t-test, and only metabolites with *P* < 0.05 were shown. POSP_H: postpartum dairy cows with high energy-corrected milk; POSP_L: postpartum dairy cows with low energy-corrected milk. Table S9. 386 differential metabolites in the plasma between POSP_H and PREP_H group (*n* = 7 per group). Data were analyzed by Student's t-test, and only metabolites with *P* < 0.05 were shown. POSP_H: postpartum dairy cows with high energy-corrected milk; PREP_H: prepartum dairy cows with high energy-corrected milk postpartum. Table S10. 385 differential metabolites in the plasma between POSP_L and PREP_L group (*n* = 7 per group). Data were analyzed by Student's t-test, and only metabolites with *P* < 0.05 were shown. POSP_L: postpartum dairy cows with low energy-corrected milk; PREP_L: prepartum dairy cows with low energy-corrected milk postpartum. Table S11. 93 differential metabolites in the milk between POSP_H and POSP_L group (*n* = 7 per group). Data were analyzed by Student's t-test, and only metabolites with *P* < 0.05 were shown. POSP_H: postpartum dairy cows with high energy-corrected milk; POSP_L: postpartum dairy cows with low energy-corrected milk. Table S12. Similarity of rumen microbial communities predicted by ANOMIS analysis (*n* = 7 per group). PREP_H: prepartum dairy cows with high energy-corrected milk postpartum; PREP_L: prepartum dairy cows with low energy-corrected milk postpartum; POSP_H: postpartum dairy cows with high energy-corrected milk; POSP_L: postpartum dairy cows with low energy-corrected milk. Table S13. Differences in KEGG enzyme-encoding genes across groups identified through metagenomic analysis (*n* = 7 per group). Data were analyzed by Kruskal-Wallis H test, and comparisons between means were undertaken using Tukey-Kramer's test. PREP_H: prepartum dairy cows with high energy-corrected milk postpartum; PREP_L: prepartum dairy cows with low energy-corrected milk postpartum; POSP_H: postpartum dairy cows with high energy-corrected milk; POSP_L: postpartum dairy cows with low energy-corrected milk.

## Data Availability

Raw sequencing data of all metagenomes have been deposited into the NCBI Sequence Read Archive (SRA) under accession numbers: PRJNA1211861 (https://www.ncbi.nlm.nih.gov/bioproject/PRJNA1211861).

## Abbreviations

A/G: Albumin to globulin ratio
ALT: Alanine aminotransferase
A/P: Acetate to propionate
BA: Bile acids
BUN: Blood urea nitrogen
CFI: Comparative fit index
ECM: Energy-corrected milk yield
IDENs: Inter-domain ecological networks
KEGG: Kyoto Encyclopedia of Genes and Genomes
RMSE: Root mean square error
TVFA: Total volatile fatty acids
NH_3_-N: Ammonia nitrogen
TMR: Total mixed ratio
VIP: Variable important for the projection
