# Supplementary material for: Multi-omics revealed the effects of rumen to blood path on early lactation performance in transition dairy cows
Source: Microbiome. 2026 Apr 7;14:146. doi: 10.1186/s40168-026-02403-y (PMC13173865; doi:10.1186/s40168-026-02403-y)
Supplement: Supplementary file 2 — Additional file 1: Fig. S1. Experimental design for this study. Healthy multiparous Chinese Holstein dairy cows (n = 100) with similar last 305-d milk yields, parity, body condition scores, and due date were selected. Ruminal fluid and blood samples of all cows were collected at −14 d (14 days before calving) and 14 d (14 days after calving) 2 hours after the morning feeding. Milk samples of all cows were collected at 14 d. Average milk yield at 13 d, 14 d, and 15 d was used to calculate the yield of energy corrected milk (ECM) of all cows. Based on the ECM yield of all cows at 14 d, the 7 cows with highest ECM yield (51.07 ± 7.76 kg/d, mean ± SD) and the 7 cows with the lowest ECM yield (31.19 ± 3.36 kg/d, mean ± SD) were used for the following analysis. The selected cows were compared based on their pregnancy status: prepartum (PREP, n = 14) and postpartum (POSP, n = 14), or based on a combination of ECM and pregnancy status: prepartum cows with high ECM postpartum (PREP_H, n = 7); prepartum cows with low ECM postpartum (PREP_L, n = 7); postpartum cows with high ECM (POSP_H, n = 7); postpartum cows with low ECM (POSP_L, n = 7). Fig. S2. Correlation analysis of ECM production with differential ruminal fermentation and plasma parameters (n = 14). In the heatmap visualization, color gradients represent correlation magnitudes, with red indicating positive correlations and blue representing negative correlations. Correlations were assessed using Spearman's correlation. * indicates q < 0.05, ** indicates q < 0.01, and *** indicates q < 0.001. ECM: energy-corrected milk yield; A/G: albumin to globulin ratio; ALT: alanine aminotransferase; A/P: acetate to propionate; BUN: blood urea nitrogen; NH3-N: ammonia nitrogen; TVFA: total volatile fatty acids. Fig S3. Identification of differential ruminal bacterial and archaeal species in high and low yield dairy cows during the transition period. A Differential bacterial species between PREP and POSP group (n = 14). B Differentia [file 40168_2026_2403_MOESM1_ESM.docx]

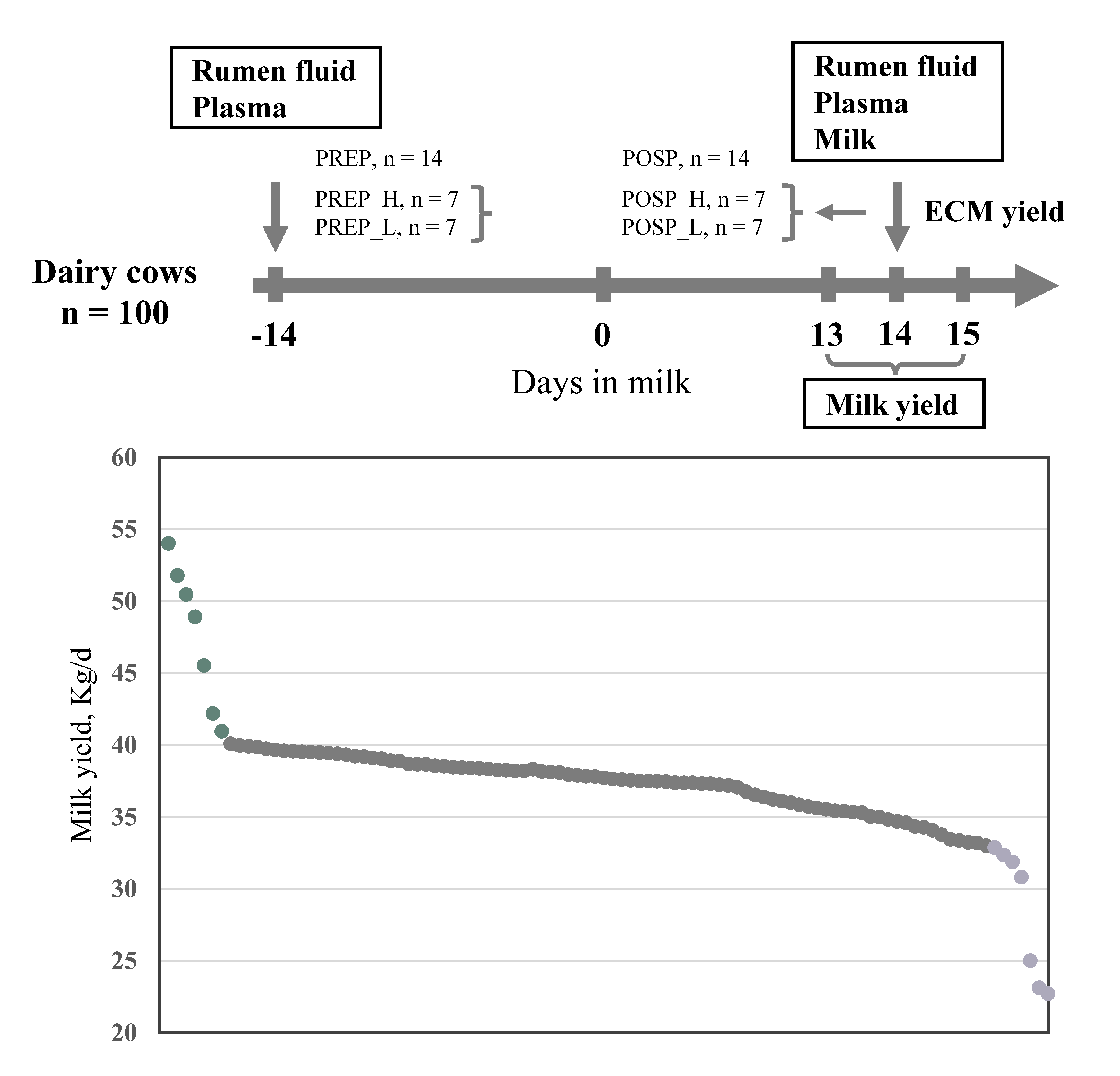


**Fig. S1 Experimental design for this study.** Healthy multiparous Chinese Holstein dairy cows (*n* = 100) with similar last 305-d milk yields, parity, body condition scores, and due date were selected. Ruminal fluid and blood samples of all cows were collected at -14 d (14 days before calving) and 14 d (14 days after calving) 2 hours after the morning feeding. Milk samples of all cows were collected at 14 d. Average milk yield at 13 d, 14 d, and 15 d was used to calculate the yield of energy corrected milk (ECM) of all cows. Based on the ECM yield of all cows at 14 d, the 7 cows with highest ECM yield (51.07 ± 7.76 kg/d, mean ± SD) and 7 cows with lowest ECM yield (31.19 ± 3.36 kg/d, mean ± SD) were used for the following analysis. The selected cows were compared based on their pregnancy status: prepartum (PREP, *n* = 14) and postpartum (POSP, *n* = 14), or based on a combination of ECM production and pregnancy status: high ECM yield cows in prepartum (PREP_H, *n* = 7); low ECM yield cows in prepartum (PREP_L, *n* = 7); high ECM yield cows in postpartum (POSP_H, *n* = 7); low ECM yield cows in postpartum (POSP_L, *n* = 7).


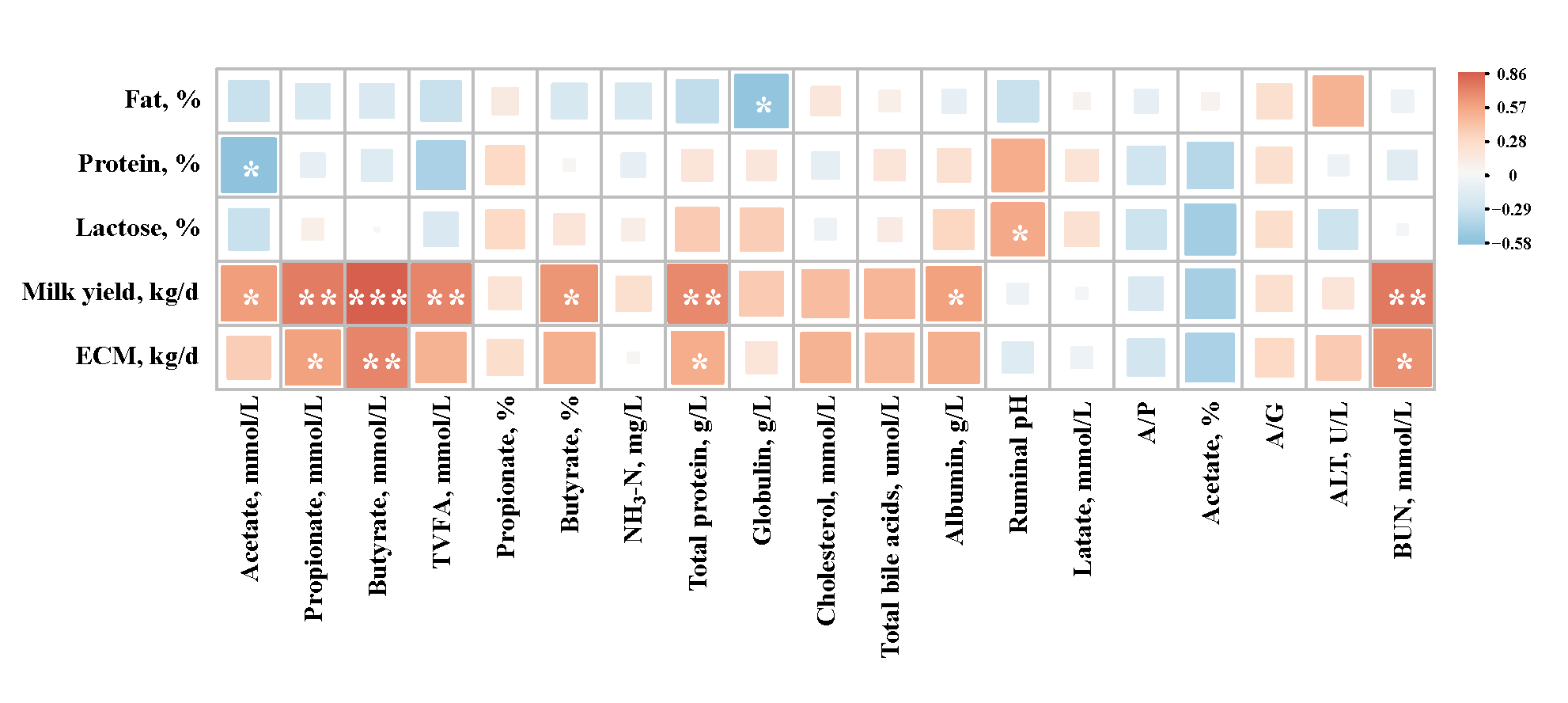


**Fig. S2 Correlation analysis of ECM production with differential ruminal fermentation and plasma parameters (*n* = 14).**

In the heatmap visualization, color gradients represent correlation magnitudes, with red indicating positive correlations and blue representing negative correlations. Correlations were assessed using Spearman's correlation. * indicates *q* < 0.05, ** indicates *q* < 0.01, and *** indicates *q* < 0.001. ECM: energy-corrected milk yield; A/G: albumin to globulin ratio; ALT: alanine aminotransferase; A/P: acetate to propionate; BUN: blood urea nitrogen; NH_3_-N: Ammonia nitrogen; TVFA: total volatile fatty acids.

**

**

**Fig S3 Identification of differential ruminal bacterial and archaeal species in high and low yield dairy cows during the transition period.**

**A** Differential bacterial species between PREP and POSP group (*n* = 14). **B** Differential bacterial species among PREP_H, PREP_L, POSP_H, and POSP_L group (*n* = 7). **C** Differential archaeal species between PREP and POSP group (*n* = 14). **D** Differential archaeal species among PREP_H, PREP_L, POSP_H, and POSP_L group (*n* = 7). Significant differences were identified by linear discriminant analysis (LDA) effect size with LDA > 2.5 and *q* < 0.05. ECM: energy-corrected milk yield; PREP_H: prepartum cows with high ECM postpartum; PREP_L: prepartum cows with low ECM postpartum; POSP_H: postpartum cows with high ECM; POSP_L: postpartum cows with low ECM.


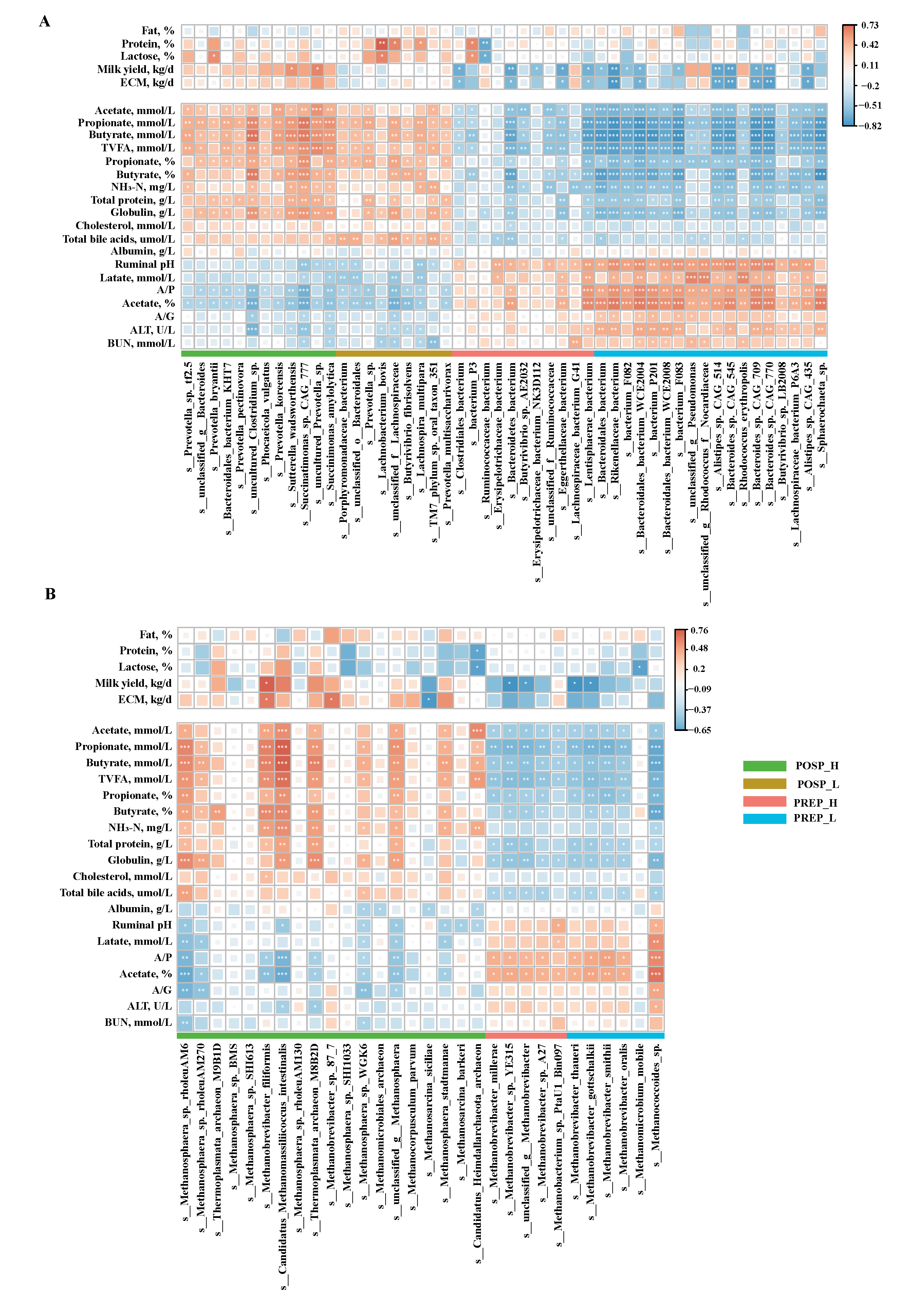


**Fig. S4 Correlation analysis between differential ruminal microbes and fermentation parameters, plasma metabolites, and energy-corrected milk yield (ECM).**

**A** Correlation analysis of differential bacterial species with ruminal fermentation (*n* = 28), plasma parameters (*n* = 28), and ECM production (*n* = 14). **B** Correlation analysis of differential archaeal species with ruminal fermentation (*n* = 28), plasma parameters (*n* = 28), and ECM yield (*n* = 14). In the heatmap visualization, color gradients represent correlation magnitudes, with red indicating positive correlations and blue representing negative correlations. In Spearman's correlation, * indicates *P* < 0.05, ** indicates *P* < 0.01, and *** indicates *P* < 0.001. A/G: albumin to globulin ratio; ALT: alanine aminotransferase; A/P: acetate to propionate; BUN: blood urea nitrogen; NH_3_-N: ammonia nitrogen; TVFA: total volatile fatty acids. PREP_H: prepartum cows with high ECM postpartum; PREP_L: prepartum cows with low ECM postpartum; POSP_H: postpartum cows with high ECM; POSP_L: postpartum cows with low ECM.


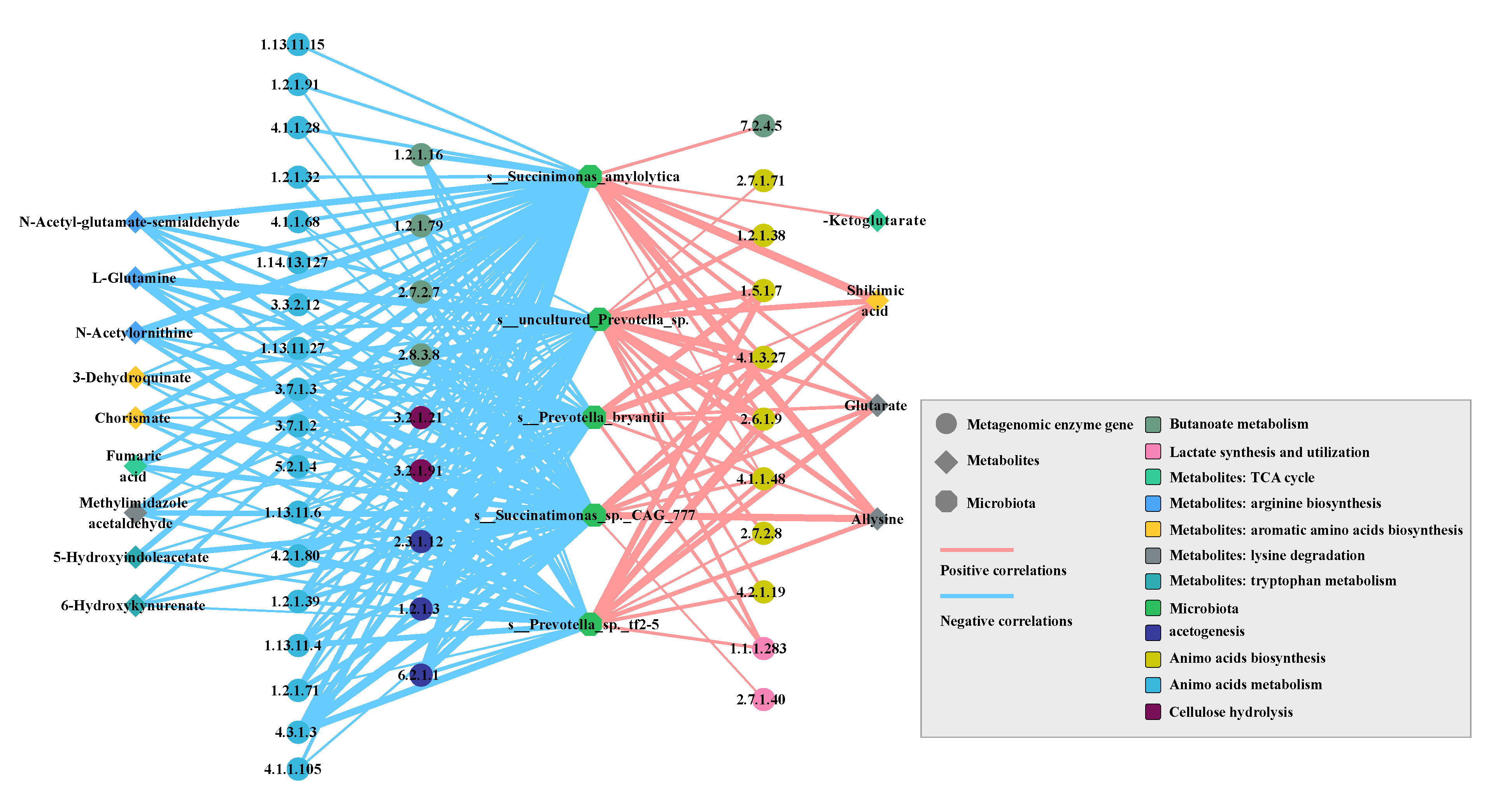


**Fig. S5 Visualization of microbial interactions with ruminal microbial functions and metabolites (*n* = 28).**

Red lines indicated positive correlations, and blue lines indicate negative correlations. The thickness of the lines represents the magnitude of the correlation coefficients. The colors of nodes indicate the group to which the species/enzyme genes/metabolites belong. Correlations were assessed using Spearman's correlation and all correlations with a *q* value < 0.05 were considered statistically significant.





**Fig S6 Pan-genomes and core-genomes of *Prevotella* (A)*,* Succinivibrionaceae (*Succinimonas* and *Succinatimonas*) (B)*,* *Methanosphaera* (C)*, Alistipes* (D), *Bacteroides* (E), and *Methanobrevibacter* (F).**

The red line represents the pan-genome size for each strain combination, while the blue line indicates the corresponding core genome size.
